# Ginsenoside CK targets PHD2 to prevent platelet adhesion and enhance blood circulation by modifying the three-dimensional arrangement of collagen

**DOI:** 10.1016/j.apsb.2024.12.038

**Published:** 2024-12-31

**Authors:** Chuanjing Cheng, Kaixin Liu, Jinling Zhang, Yanqi Han, Tiejun Zhang, Yuanyuan Hou, Gang Bai

**Affiliations:** a State Key Laboratory of Medicinal Chemical Biology, College of Pharmacy and Tianjin Key Laboratory of Molecular Drug Research, Nankai University, Tianjin 300353, China; b State Key Laboratory of Drug Delivery Technology and Pharmacokinetics, Tianjin Key Laboratory of Quality Markers of Traditional Chinese Medicine, Tianjin Institute of Pharmaceutical Research, Tianjin 300462, China

**Keywords:** PHD2, Platelet adhesion, Collagen, PHD2 inhibitor, Ginsenoside CK, Blood circulation, Thrombotic diseases, Von willebrand factor

## Abstract

Platelets are indispensable for physiological hemostasis and pathological thrombus formation, and platelet adhesion to endothelial collagen is a critical initial step in thrombus formation, often overlooked in current antiplatelet therapies. This study aims to elucidate how ginsenoside CK enhances hemodynamic circulation, alleviates stasis, and proposes therapeutic mechanisms. Inspired by the effects on improving microcirculatory disturbances in an acute soft tissue injury model, CK was identified as a PHD2 inhibitor, effectively suppressing platelet adhesion to collagen. It was proposed that targeting PHD2 regulates collagen hydroxylation modification, thereby influencing the formation of its three-dimensional structure, reducing the binding affinity between VWF and collagen, and ultimately suppressing thrombotic events. The efficacy of this mechanism was subsequently confirmed through a mouse DIC model, demonstrating the feasibility of CK in alleviating circulatory disorders. It is worth noting that when *Phd2* was knocked down in mice's lungs, pulmonary embolism was significantly reduced. Additionally, PHD2 inhibitors approved for other diseases have exhibited similar anti-thrombotic effects. Moreover, when PHD2 inhibitors were combined with aspirin, they more effectively inhibited arterial thrombosis in rats. The findings offer valuable insights into potential targets for developing antiplatelet drugs or expanding therapeutic applications for existing PHD2 inhibitors in treating thrombotic diseases.

## Graphical abstract

Ginsenoside CK targets PHD2 to reduce platelet adhesion and enhance blood circulation by modulating the three-dimensional structure of collagen, providing insights for therapies targeting thrombotic diseases.

**Figure:**
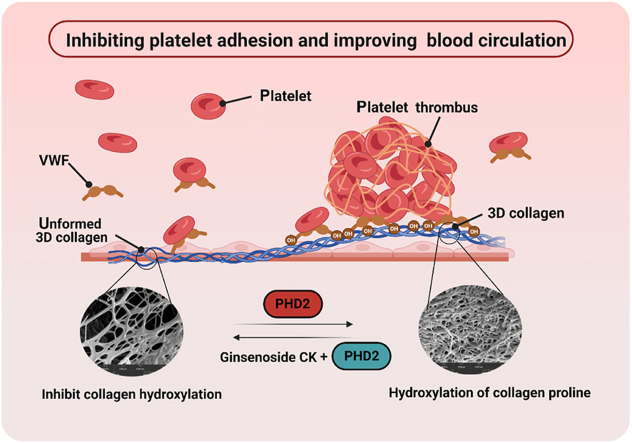

## 1 Introduction

The global mortality rate for thromboembolic diseases is about 25%. Compared to arterial thrombosis, such as in heart disease and stroke, public awareness of venous thromboembolism (including pulmonary embolism and deep vein thrombosis) is relatively low worldwide^1^. Currently, platelet thrombus inhibitors primarily encompass compounds that inhibit platelet activation and aggregation, regulate the coagulation cascade, and incorporate fibrinolytic agents into other components^2^. The constant demand for novel platelet thrombus inhibitors stems from concerns regarding the risks of bleeding, potential drug interactions, or resistance issues associated with numerous existing drugs.

Collagen, the predominant protein in the extracellular matrix, plays a crucial role in platelet thrombosis by serving as a binding site for platelets during blood vessel injuries. Following vascular wall injury, the interaction between platelets and subendothelial collagen is the pivotal initial step in thrombus formation^3^. At moderate to high shear rates, von Willebrand factor (VWF) binds platelet receptor glycoprotein (GP) Ib-IX-V, tethering platelets to VWF bound on collagen^4^. Subsequent stable platelet adhesion and activation on collagen involve two collagen receptors, namely, immunoglobulin-like receptor GPVI and integrin *α*2*β*1. Studies have demonstrated that thrombus formation on collagen/VWF relies on the synergistic interaction between these receptors, with GPIb cooperating with *α*2*β*1 or GPVI to mediate adhesion, while *α*2*β*1 enhances signal transduction through GPVI, leading to platelet activation^5^, ^6^, ^7^. The release of self-secreted mediators like ADP and thromboxane A2 plays a critical supportive role in platelet recruitment and capture. Subsequently, this triggers platelet procoagulant responses, strongly promoting localized thrombin generation and establishing the coagulation machinery, facilitating thrombus formation^8^.

There is substantial evidence indicating the significant role of VWF-dependent platelet activation in the progression of thrombotic syndromes. Indeed, it has been demonstrated that disrupting the collagen–VWF–GPIb/V/IX interaction is a promising anti-thrombotic strategy, potentially safer than blocking platelet aggregation^9^. Monoclonal antibodies and other compounds interfering with the VWF–GPIb axis have shown considerable antithrombotic potential in animal models^10^^,^^11^. These studies have confirmed the anticipated potent antithrombotic effects, with a broad therapeutic window and minimal impact on bleeding time. However, the hindrance to development lies in their inability to be administered orally.

Additionally, VWF can interact with exposed subendothelial prolyl hydroxylated collagen through its A1 and A3 domain^12^. Subsequently, VWF undergoes conformational changes, allowing it to interact with platelet receptor GPIb*α* and mediate platelet adhesion. Prolyl hydroxylases (PHDs) belong to 2-oxoglutarate-dependent dioxygenases^13^. Due to their involvement in regulating hypoxia-inducible factor (HIF) signaling, they have garnered widespread attention as potential therapeutic targets for various diseases, including anemia, ischemic heart disease, stroke, cancer, and pulmonary arterial hypertension^14^^,^^15^. Undoubtedly, the hydroxylation of proline residues mediated by PHDs plays a crucial role in maintaining the structural integrity of collagen's three-dimensional framework, while collagen cross-linking significantly contributes to platelet adhesion during thrombosis. However, this aspect is currently overlooked in the development of antiplatelet thrombotic drugs.

The elucidation of the interaction between active molecules and targets and the process of information transmission based on traditional Chinese medicine (TCM) theory represents a practice research pathway. It can facilitate a profound understanding of disease pathogenesis and establish a clear intervention strategy for disease treatment^16^. TCM is widely employed for promoting blood circulation, eliminating blood stasis, reducing swelling, and alleviating pain. A growing body of research, such as *Salvia miltiorrhiza* and *Panax ginseng*, focused on ameliorating blood circulation disorders and preventing/treating blood clots^17^^,^^18^. Studies have shown that it can prevent platelet aggregation, inhibit coagulation cascade, inhibit inflammation, and prevent monocyte attack to explain its mechanism of action^19^. *Panax notoginseng*, Chinese name Sanqi, renowned for its hemostatic and analgesic properties, is widely recognized as a TCM utilized to enhance blood circulation and alleviate blood stasis. Its extracts have been extensively studied in modern pharmacology, revealing diverse functions, such as protection against cerebrovascular injury, cardioprotective effects, hemostasis and anticoagulation, and antitumor effects^20^, ^21^, ^22^. Despite these findings, the specific targets and regulatory mechanisms underlying the blood-activating effects remain incompletely understood.

Herein, building upon previous findings^23^, we selected ginsenoside CK (CK), an active metabolite of ginsenosides in Sanqi^24^, as our research subject to investigate its therapeutic mechanisms. Initially, we assessed its efficacy in blood activation using a mechanical impact-induced rat acute soft tissue injury model or mouse disseminated intravascular coagulation (DIC) model. By conducting metabolomics analysis, drug affinity responsive target stability (DARTS) evaluation, and utilizing target-silencing animal models, we have elucidated CK's potential target protein PHD2. Furthermore, we employed various chemical biology techniques to investigate the mechanisms underlying the inhibition of proline hydroxylation in collagen and its regulatory actions in platelet thrombus formation.

## 2 Materials and methods

### 2.1 Materials and reagents

Ginsenoside CK (B21045; HPLC≥98%) and *N*-acetyl-l-cysteine (NAC, S20137-25g) were procured from Shanghai Yuanye Biotechnology Co., Ltd. (Shanghai, China). Ibuprofen (IBU, Q108755-5g) was sourced from Shanghai Dibai Biotechnology Co., Ltd. (Shanghai, China). IOX2 (HY-15468), roxadustat (Rox, HY-13426), vadadustat (Vad, HY-101277) and daprodustat (Dap, HY-17608) were purchased from Med Chem Express (NJ, USA). Calcein-AM (C832705) and FeCl_3_ (I811935) were purchased from Shanghai Macklin Biochemical Technology Co., Ltd. (Shanghai, China) and type I rattail tendon collagen and aspirin (ASP, A8830) were supplied by Beijing Solarbio Co. (Beijing, China). Antibodies targeting PHD2 (4835S), HIF-1*α* (48085S), VEGF (9698S), collagen I (72026S), and GAPDH (5174S) were obtained from Cell Signaling Technology (Boston, USA). Anti-fibrinogen antibody (bs-7548R) was purchased from Beijing Bioss Biotechnology Co., Ltd. (Beijing, China). And rabbit polyclonal antibody to thrombin (#AF0357) was got from Affinity Biosciences (Beijing, China), VWF monoclonal antibody (66682-1-Ig) was provided by Protein tech Group, Inc. (Wuhan, China). Goat anti-rabbit IgG H&L (Alexa Fluor® 594, ab150080) and HRP anti-rabbit IgG antibody (ab288151) were acquired from Abcam (Cambridge, UK).

### 2.2 Acute soft tissue injury in rats

Acute soft tissue injury was induced in male SD rats (200–220 g) using mechanical stress methodology with modifications as previously described^23^. Rats were randomly divided into five groups: control (con), model (mod), positive control (NAC, 70 mg/kg), and CK treatment groups (12.5 and 25 mg/kg). Three days prior to injury, NAC or CK was administered *via* intraperitoneal injection to the rats. Following a 12-h fasting period, rats were anesthetized with 2% sodium pentobarbital. A stainless-steel hammer weighing 100 g was dropped from a height of 100 cm, striking the middle part of the right hind leg muscle five times. Leg swelling values were recorded 24 h after injury. Continued dosing for 4 days was performed, and peripheral blood and injured muscle tissue were collected for subsequent measurements of hemorheology, blood viscosity, and pathological sections. Animal experiments were performed following the National Institute of Health Guide for the Care and Use of Laboratory Animals, and all procedures were approved by the Tianjin Institute of Pharmaceutical Research (Registration number: 2020080303, date: 03/08/2020).

### 2.3 LPS-induced mouse DIC model

In essence, this research utilized ICR mice weighing approximately 25 g, and the DIC model was meticulously constructed following established protocols^25^. Subsequently, CK (10, 20, 40 mg/kg), PHD2 inhibitor (Dap, 10 mg/kg) or positive control ASP (10 mg/kg) was injected intraperitoneally.

Following model construction, mice were anesthetized through intraperitoneal injection of pentobarbital sodium. Subsequently, their tails were incised 3 mm from the tip, and the mouse tails were promptly immersed vertically in physiological saline at 37 °C to record tail bleeding duration and measure bleeding volume, assessed *via* absorbance readings. Subsequently, lung tissues from the mice were meticulously sectioned for histological staining and subsequent analysis. Animal experiments were performed following the National Institute of Health Guide for the Care and Use of Laboratory Animals, and all procedures were approved by the Medicine Institutional Animal Ethics and Welfare Committee of Nankai University (Registration number: 2022-SYDWLL-000023, date: 28/02/2022).

### 2.4 FeCl3-induced rat arterial thrombosis

SD rats (200–220 g) were randomly divided into seven groups: control (ctrl), model (mod), ASP (10 mg/kg), CK (20 mg/kg), Dap (10 mg/kg), ASP + CK (5 + 10 mg/kg), and ASP + Dap (5 + 5 mg/kg) groups. Continuous intraperitoneal administration of drugs was performed for 14 days. Anesthesia was induced in rats 1 h after the final administration, and blunt dissection to isolate the carotid artery. Thrombus formation was induced by placing a filter paper (10 mm × 10 mm) soaked in 35% FeCl_3_ on the right carotid artery for 10 min. After 60 min, blood was collected into sodium citrate tubes for subsequent coagulation parameter measurements. Subsequently, the carotid arteries were collected, and thrombus length and wet weight were measured, and subjected to histopathological staining analysis.

### 2.5 Nontargeted metabolomics

Plasma obtained from rats with soft tissue injuries was utilized for high-resolution untargeted metabolomics analysis. The technical expertise for metabolomics analysis and identification was provided by Shanghai Zhongke New Life Biotechnology Co., Ltd. (Shanghai, China). The LC–MS analysis method and metabolite identification process are detailed in Supporting Information (Supporting Information Fig. S1). Significantly different metabolites were selected based on a VIP value > 1 and *P* value < 0.05. Subsequently, the impacted metabolic pathways were further investigated through differential metabolite screening, differential metabolite correlation analysis, and KEGG pathway enrichment analysis.

### 2.6 Interaction analysis techniques

In this study, a variety of methods were employed to analyze protein-small molecule and protein–protein interactions, including surface plasmon resonance (SPR), microscale thermophoresis (MST), and fluorescence thermal shift assay (FTSA). Detailed experimental conditions can be found in the Supporting Information

### 2.7 Scanning electron microscopy (SEM)

The microscopic structure of collagen protein was examined through scanning electron microscopy. Isolated collagen protein was directly adhered onto conductive adhesive, and coated with a thin layer of gold using the Quorum SC7620 sputter coater for 45 s with a sputtering current of 10 mA. Subsequently, the sample morphology was captured using the TESCAN MIRA LMS scanning electron microscope^26^.

### 2.8 Peptide mass spectrometry

The collagen *α*1 chain bands were excised from the SDS-PAGE gel and subjected to in-gel trypsin digestion. Trypsin-digested peptides were analyzed using liquid chromatography (LC) coupled with an LCQ Deca XP ion trap mass spectrometer equipped with electrospray ionization (Thermo Finnigan). A C_8_ capillary column was used with a flow rate of 4.5 μL/min and an LC mobile phase consisting of buffer A (0.1% formic acid in MilliQ water) and buffer B (0.1% formic acid in 3:1 acetonitrile: isopropanol, *v*/*v*). The LC sample flow was introduced into the mass spectrometer *via* the electrospray ionization source with a spray voltage of 3 kV. Peptide identification was performed using Sequest search software (Thermo Finnigan) against the NCBI protein database. For large collagen peptides, manual identification was required by calculating potential MS/MS ions and subsequently matching them to the corresponding MS/MS spectra^27^.

### 2.9 Platelet adhesion and collagen binding

Platelets (2 × 10^−7^ cells/mL) were seeded onto glass coverslips coated with 10 μg/mL collagen (incubated overnight at 4 °C) and incubated at 37 °C for 90 min. After washing with PBS, platelets were fixed, permeabilized, stained with Calcein-AM, and observed using a fluorescence microscope (Leica TCS SP8, Germany). Quantitative analysis of platelet adhesion on collagen was performed by Image J software. Additionally, 96-well microplates were coated overnight with type I collagen, followed by the addition of rat plasma into the wells, and collagen binding of VWF was detected using VWF antibodies.

### 2.10 Molecular docking

AutoDock Vina 1.1.2 software was used for molecular docking. The 3D structure of PHD2 (PDB ID: 2g19) was obtained from the RCSB Protein Database (www.rcsb.org), referring to the previous docking method and using PyMOL 2.3.2 for image processing^28^.

### 2.11 Construction of Phd2 knockdown mouse model

The sh-*Phd2* plasmid (target sequence: 5′-AGACTGGGACGCCAAGGTA-3′) was purchased from Vigene Biosciences, Inc. (Shandong, China). In summary, sh-*Phd2* or a negative control sequence was inserted into the vector (pAV-U6-shRNA-CMV-Intron-mCherry). Subsequently, the recombinant plasmid carrying the sh-*Phd2* gene was co-transfected with helper plasmids into HEK293T packaging cells. After 72 h of transfection, a significant amount of recombinant virus was produced within the cells. And 20 μL (2.5 × 10^−13^ vg/mL) of AAV9 expressing sh-*Phd2* or a negative control (sh-con) was administered into the mouse trachea and maintained for 2 weeks^29^. The efficiency of PHD2 knockdown in the lungs was assessed by Western blotting.

### 2.12 Statistical analysis

In this study, the data are shown as mean ± standard error of mean (SEM). Student's *t*-test compared two datasets, and one-way analysis of variance compares multiple datasets, including Dunnett's and Tukey's multiple comparisons test. Data analysis utilized GraphPad Prism 6.01 software (La Jolla, CA, USA). A significance level of *P* < 0.05 indicated statistical significance.

## 3 Results

### 3.1 CK improves acute soft tissue injuries in rats

**Figure 1:**
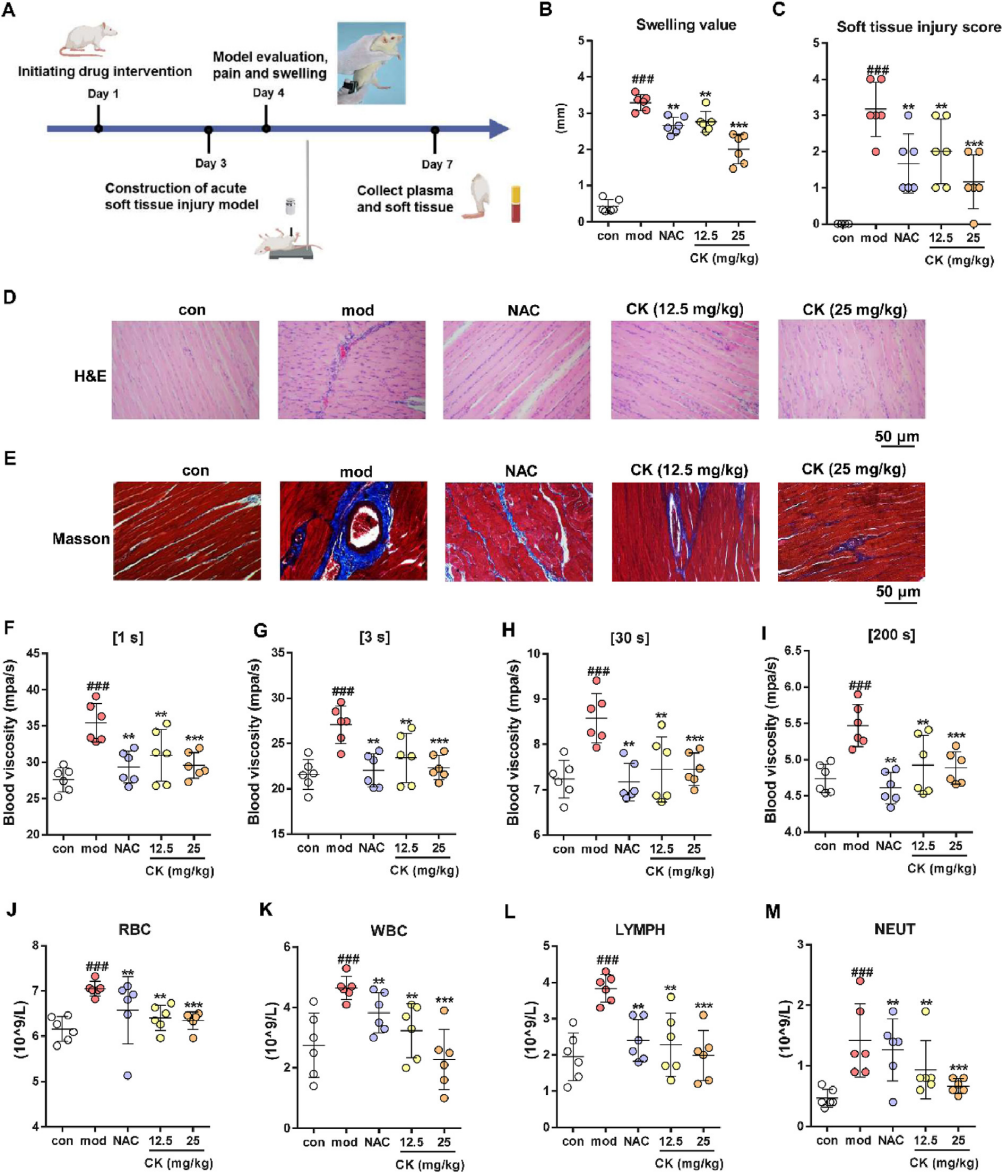
CK ameliorates acute soft tissue injuries in rats. (A) Schematic representation of the construction process for rat soft tissue injury model. (B) Measurement of swelling at the injury site. (C) Soft tissue injury score statistics and H&E (D) and Masson (E) staining analysis of soft tissue. (F–I) Determination of whole blood viscosity under low (1 and 3 s), medium (30 s), and high (200 s) shear conditions. RBC (J), WBC (K), LYMPH (L), and NEUT (M) counts in rats with soft tissue injuries. ^###^*P* < 0.001 *vs*. con group; ∗∗*P* < 0.01, ∗∗∗*P* < 0.001 *vs*. mod group, (*n* = 6).

Acute soft tissue injury is characterized by local microcirculation disorders, resulting in impaired blood flow and increased blood viscosity. In order to explore the potential mechanism of CK, a rat model of acute soft tissue injury was employed. The multifunctional antioxidant NAC was selected as a positive control in the rat model of acute soft tissue injury characterized by local hypoxia^30^. Drug administration and treatment were conducted according to the experimental procedure outlined in Fig. 1A. The results showed that the mod group exhibited a significant increase in swelling compared to the con group, whereas CK (12.5 and 25 mg/kg) treatment demonstrated efficacy in reducing tissue edema (Fig. 1B). Furthermore, an integrated score for the injured site also indicated that CK treatment significantly ameliorated tissue damage (Fig. 1C). The injured soft tissue sections were subsequently observed using H&E and Masson staining (Fig. 1D and E). Compared to the mod group, the CK and NAC groups exhibited alleviation of collagen fiber proliferation, irregular arrangement or necrosis of muscle fibers, as well as bleeding and severe blood stasis.

Soft tissue injuries result in the accumulation of vasoactive substances, prostaglandins, and other inflammatory mediators at the site of damage, leading to a cascade of microcirculation disturbance. Experimental results (Fig. 1F–I) demonstrated a significant increase in whole blood viscosity under different shear conditions after molding. However, treatment with NAC and CK exhibited varying degrees of improvement. As expected, the plasma of the mod group exhibited significant elevations in red blood cell (RBC) count, white blood cell (WBC) count, lymphocytes (LYMPH), and neutrophils (NEUT) (Fig. 1J–M), indicating the presence of inflammation and tissue damage. These counts demonstrated varying degrees of reduction following the intervention. In summary, the analysis above indicates that CK significantly ameliorates blood circulation disorders and mitigates inflammatory damage caused by soft tissue injuries in rats.

### 3.2 CK affects HIF-1 signaling pathway

**Figure 2:**
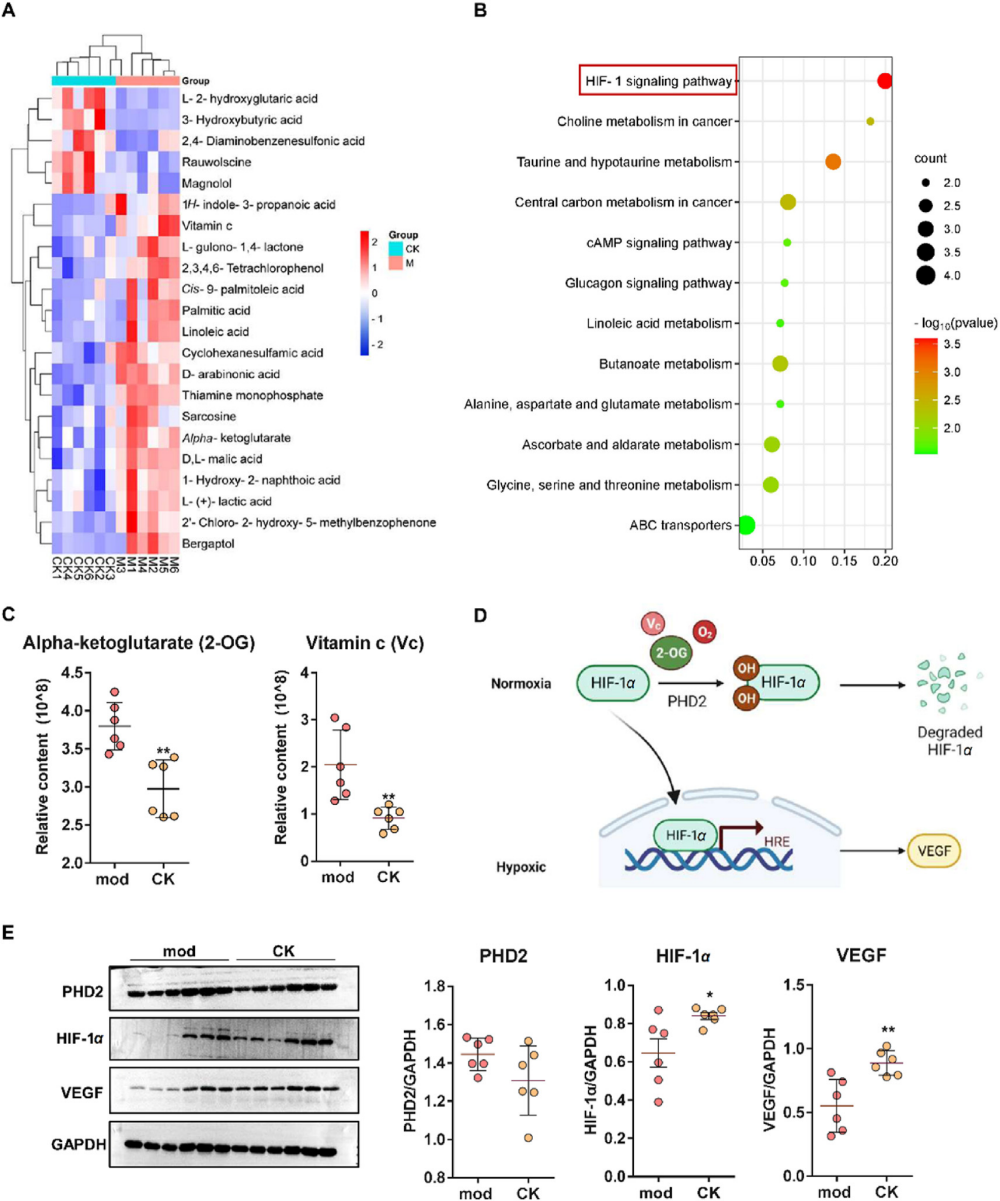
Untargeted plasma metabolomics analysis and evaluation on rat soft tissue injury. (A) Cluster analysis of significantly differential metabolites affected by CK. (B) Enrichment analysis of metabolic pathways based on significantly differential metabolites. (C) The impact of CK on the key metabolites 2-OG and Vc in HIF-1 signaling pathway. (D) Schematic diagram of the role of PHD2 and its associated metabolites in the HIF-1*α* signaling pathway. (E) Western blot detection of the effect of CK on the expression of PHD2, HIF-1*α* and VEGF. ∗*P* < 0.05, ∗∗*P* < 0.01 *vs*. mod group, (*n* = 6).

Untargeted plasma metabolomics was used to analyze the metabolites and related pathways affected by CK *in vivo*. After filtering, 22 significantly different metabolites were found, and cluster analysis was performed with a heat map (Fig. 2A). The functions and enrichment levels of different metabolites were analyzed according to KEGG metabolic pathways. The results showed that CK mainly acts on the HIF-1 signaling pathway, choline metabolism, taurine and hypotaurine metabolism (Fig. 2B). In a hypoxic environment, after implementing CK intervention, Fig. 2C reveals two significant differential metabolites, namely, alpha-ketoglutarate (2-oxoglutarate, 2-OG) and vitamin C (Vc). These metabolites are intricately linked to the PHD2 in regulating hydroxylation modifications of HIF-1*α*. Hypoxia allows HIF-1*α* to escape ubiquitination and degradation, thereby activating its entry into the nucleus and initiating the transcriptional expression of genes including VEGF. Targeted inhibition of PHD2 promotes HIF-1*α* activation and increases VEGF expression (Fig. 2D). Subsequently, we evaluated the expression of key proteins in the HIF-1 signaling pathway in injured soft tissue. As shown in Fig. 2E, the expression of PHD2 protein remains unaffected by CK; however, the CK treatment group exhibits an increased expression of HIF-1*α* and its downstream protein marker VEGF. The data presented above demonstrated the potential correlation between CK and the HIF-1 signaling pathway in inhibiting PHD2 function within the context of soft tissue injuries.

### 3.3 CK targets PHD2 protein

**Figure 3:**
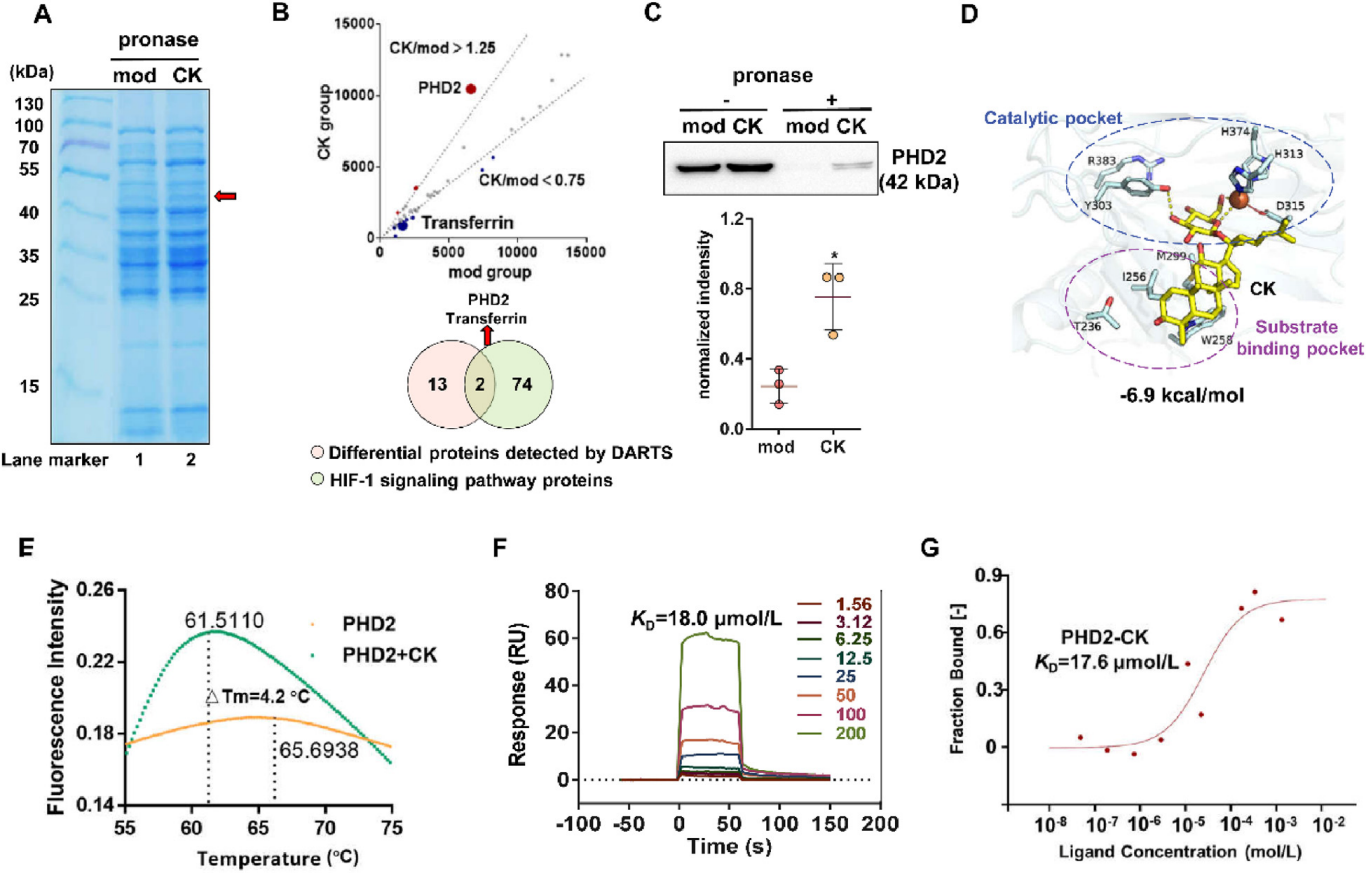
PHD2 protein is identified as a target of CK. (A) DARTS assay of CK's effect on damaged soft tissue proteins on SDS-PAGE. (B) Identification of potential target proteins of CK by HPLC–MS/MS analysis. (C) Western blot of PHD2 target proteins on DARTS samples. ∗*P* < 0.05 *vs*. mod group (*n* = 3). (D) Molecular docking of CK molecules and PHD2 protein. (E) FTSA analysis of PHD2 protein treated with or without 50 μmol/L CK. (F) SPR analysis of CK and PHD2 (*K*_D_ = 18.0 μmol/L). (G) MST analysis of CK and PHD2 (*K*_D_ = 17.6 μmol/L).

To explore potential targets of CK, the DARTS assay was performed following previously reported methods^23^. Analysis of pronase-digested protein bands using SDS-PAGE revealed distinct differences between the CK and model group in the soft tissue injury rats (Fig. 3A). Additionally, the proteins were considered differential based on HPLC–MS/MS analysis if their CK/mod ratio was below 0.75 (blue dots) or above 1.25 (red dots). A total of 15 differentially expressed proteins were identified (Fig. 3B). Then a joint analysis was conducted with HIF-1 signaling pathway-associated proteins, among them PHD2 (with a ratio of 1.59) and transferrin (with a ratio of 0.56) are the most likely candidates for CK's target proteins. Given that transferrin primarily participates in the transport of iron ions in the plasma, our focus shifted towards investigating the interaction between PHD2 and CK, verified by Western blot analysis (Fig. 3C). And molecular docking analysis indicated that CK has the capability to occupy both the catalytic pocket and the substrate binding pocket of the PHD2 protein concurrently, displaying a binding energy of −6.9 kcal/mol (Fig. 3D).

Then we expressed and purified the PHD2 protein (Supporting Information Fig. S2) to assess the binding affinity between PHD2 and CK. FTSA analysis demonstrated that 50 μmol/L CK increased the melting temperature of PHD2 by 4.2 °C (Δ*T*_m_) (Fig. 3E). SPR experiments revealed that CK bound to immobilized PHD2 protein with a dissociation constant (*K*_D_) of 18.0 μmol/L (Fig. 3F). Furthermore, Fig. 3G shows that CK and PHD2 have a binding affinity of about 17.6 μmol/L by MST analysis. These findings unequivocally established that CK specifically targets PHD2, resulting in conformational changes in its structural stability.

### 3.4 CK affects collagen structure by inhibiting proline hydroxylase

**Figure 4:**
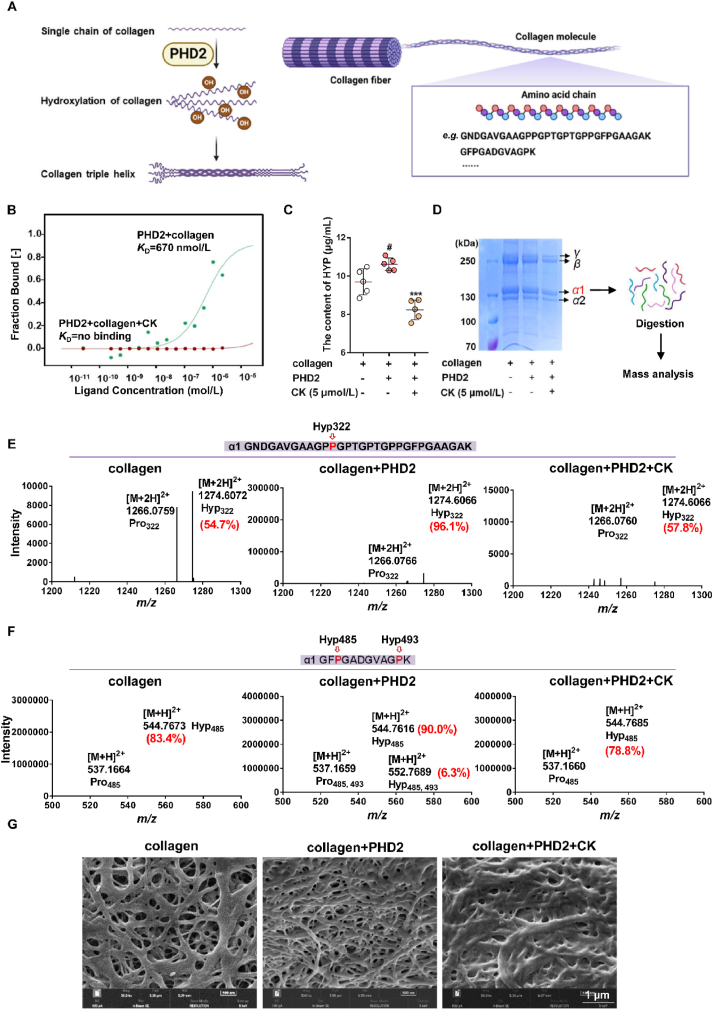
CK affects collagen structure by inhibiting prolyl hydroxylase. (A) Schematic diagram of hydroxylation modification of collagen structure by PHD2. (B) MST analysis of CK on PHD2–collage interaction. (C) Effect of CK on HYP content in collagen. ^#^*P* < 0.05 *vs*. collagen group; ∗∗∗*P* < 0.001 *vs*. PHD2 group (*n* = 5). (D) Protein profile identification flow chart of collagen *α*1 chain. The effect of CK on the hydroxylation ratio (E) or hydroxylation number (F) of representative collagen *α*1 peptides. (G) SEM imaging analysis of architecture for collagen with or without PHD2 or CK administration.

The collagen molecules are made up of three polypeptide chains that have a repeat sequence of Gly–X–Y amino acids. These chains intertwine to form a stable triple helix structure catalyzed by proline hydroxylase, including PHD2. The assembling process of collagen fiber was illustrated *via* the schematic diagram in Fig. 4A. To assess the impact of CK on collagen structure, the binding affinity between collagen and PHD2 was initially measured by MST at about *K*_D_ = 670 nmol/L. And the interaction can be destroyed by the CK administration (Fig. 4B). Fig. 4C illustrates the quantification of hydroxyproline (HYP) content in collagen. In the presence of PHD2, HYP content was increased, while pretreatment with 5 μmol/L CK inhibited HYP formation.

Additionally, protein profiling was performed on the representative *α*1 chain in collagen, separated by SDS-PAGE (Fig. 4D). Following in-gel enzymatic hydrolysis, the hydroxylation modifications and information regarding representative peptides were detected. The results demonstrate that PHD2 could catalyze an increase in the hydroxylation ratio (Fig. 4E) or the number of hydroxylation (Fig. 4F), while CK treatment attenuated the formation of hydroxylation modifications in collagen.

To visualize the alterations in collagen structure, a SEM analysis of collagen was performed. The evidences in Fig. 4G demonstrates that the natural collagen possesses a soft, multi-layered sponge-like structure characterized by a loose, fibrous, and porous microstructure. Upon being modified by PHD2, the collagen structure became denser due to reduced fiber diameter and enhanced network interconnectivity. Furthermore, treatment with CK effectively changed the stable architecture of collagen.

### 3.5 CK reduces platelet adhesion by disrupting the binding between VWF and collagen

**Figure 5:**
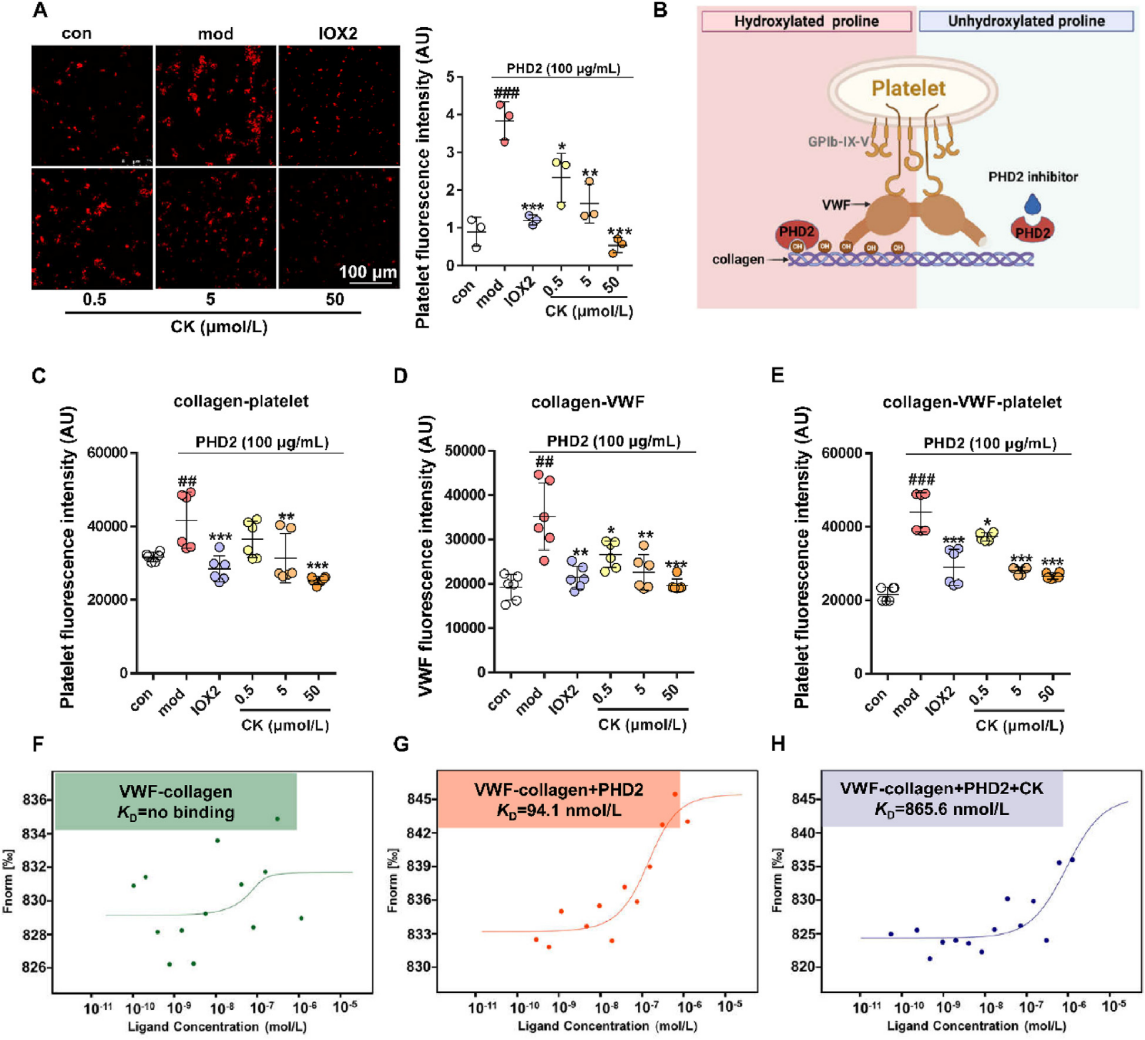
CK reduces platelet adhesion and disrupts the binding between VWF and collagen. (A) Effect of CK on fluorescence imaging of platelet adhesion with collagen protein which coated on microporous plates. ^###^*P* < 0.001 *vs*. con group; ∗*P* < 0.05, ∗∗*P* < 0.01, ∗∗∗*P* < 0.001 *vs*. mod group (*n* = 3). (B) VWF-collagen interaction diagram. CK affects PHD2 mediated (C) collagen–platelet adhesion (D) collagen–VWF binding, and (E) the adhesion of collagen–VWF-platelet. ^###^*P* < 0.001 *vs*. con group; ∗*P* < 0.05, ∗∗*P* < 0.01, ∗∗∗*P* < 0.001 *vs*. mod group (*n* = 6). (F) MST analysis of VWF and collagen. (G) The impact of PHD2 on VWF–collagen interaction. (H) The effect of CK on the interaction between VWF and collagen.

Collagen exposed on blood vessel surfaces significantly enhances platelet adhesion and facilitates thrombus formation under blood flow conditions. After investigating the impact of CK on collagen structure through PHD2-mediated proline hydroxylation, we proceeded to evaluate its influence on platelet adhesion to collagen. As depicted in Fig. 5A, our findings indicated that following the interaction between PHD2 and collagen, a significant number of platelets adhered to the substrate and formed aggregates on microporous plates. However, treatment with CK or PHD2 inhibitor IOX2 noticeably impaired platelet spreading and inter-platelet interactions on collagen.

The GPIb–IX–V complex on platelets adheres to collagen fibers facilitated by the presence of VWF factor, constituting the primary mechanism for platelet adhesion onto the collagen surface. This process is intricately linked to collagen hydroxylation mediated by PHD2 (Fig. 5B). To elucidate the pivotal role of CK in platelet adhesion to collagen, interaction tests were conducted between VWF and collagen coated with microporous plates. The inhibitory effect of CK on platelet adhesion was confirmed by fluorescence indicated antibody detection of collagen-bound platelets (Fig. 5C) and then collagen-bound VWF (Fig. 5D). It was revealed that PHD2-mediated collagen hydroxylation enhances the binding affinity, while treatment with CK disrupts the interaction between VWF and collagen, consequently attenuating platelet adhesion to collagen (Fig. 5E).

Additionally, we employed the MST technique further to evaluate the adhesive strength between VWF and collagen. As illustrated in Fig. 5F, it is evident that VWF demonstrates minimal direct binding to collagen. However, when modified with PHD2, proline hydroxylated collagen has a strong affinity for VWF emerges at *K*_D_ = 94.1 nmol/L (Fig. 5G). Conversely, CK significantly reduces the adhesive force between these entities by almost ten times to *K*_D_ = 865.6 nmol/L (Fig. 5H). These findings validate that CK effectively hinders the interaction between VWF and collagen, ultimately obstructing platelet adhesion.

### 3.6 CK inhibits collagen proline hydroxylation to improve blood circulation in rats

**Figure 6:**
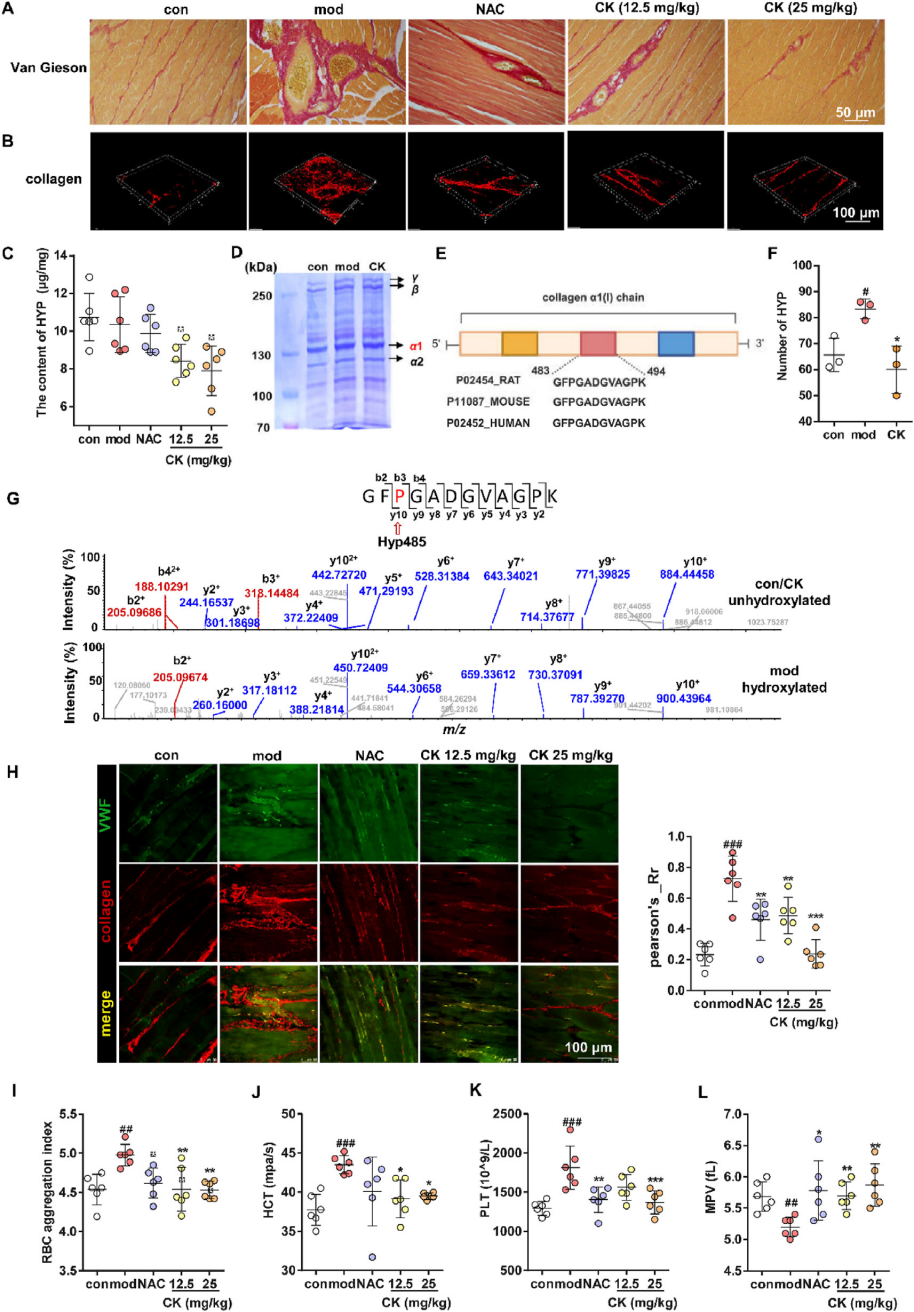
CK improves blood circulation by changing collagen structure in soft tissue injury rats. (A) Van Gieson staining for collagen on damaged soft tissues. (B) Fluorescence imaging of exposed 3D structure of collagen on soft tissue slices using antibody fluorescence detection. (C) The impact of CK on collagen HYP content. (D) Extraction and SDS-PAGE separation of collagen protein. (E) Sequence alignment of representative peptide segments of collagen *α*1 chain among rat, mouse and human. (F) Protein profile assay for the impact of CK on the total hydroxylation modification number in rat soft tissues. (G) Identification of representative peptide MS/MS spectra for prolyl hydroxylation modification. (H) Immunohistochemical analysis of VWF-collagen on injured soft tissues. The effect of CK on blood parameters in soft tissue injury rats, including RBC aggregation index (I), HCT (J), PLT (K) and MPV (L). ^#^*P* < 0.05, ^##^*P* < 0.01, ^###^*P* < 0.001 *vs*. con group; ∗*P* < 0.05, ∗∗*P* < 0.01, ∗∗∗*P* < 0.001 *vs*. mod group (*n* = 6).

Subsequently, we elucidated the mechanism underlying blood circulation regulation by targeting PHD2 in a rat model of soft tissue injury. To confirm the presence of damaged soft tissues, Van Gieson staining was employed to reveal severe bleeding with blood cell aggregation, substantial accumulation of collagen fibers (red), and disorganized muscle fiber bundles (yellow) in Fig. 6A. Conversely, significant improvements were observed in the groups treated with NAC or CK. Afterward, a fluorescence microscope was used to capture the 3D structure of exposed collagen in tissue. As shown in Fig. 6B, the mod group displayed a more distinct exposure of collagen, forming a complex network structure around ruptured blood vessels compared to the con group. Notably, this effect was effectively attenuated by the administration of NAC or CK.

The analysis of HYP content in the soft tissues showed no significant difference between mod and con groups. Nevertheless, treatment with CK led to a noticeable reduction in collagen hydroxylation. It was due to CK's inhibitory effect on PHD2, which consequently resulted in changes in collagen (Fig. 6C). To validate this hypothesis, type I collagen was extracted from injured soft tissue following the Birkedal-Hansen method^31^. The primary collagen bands and *α*1 chain were obtained from SDS-PAGE (Fig. 6D). The alignment analysis demonstrated that the *α*1 chain has a high sequence homology across species (Rat, Mouse and Human) (Fig. 6E). The protein mass spectroscopy was carried out to confirm the hydroxylation differences among each enzymatic hydrolysis group. Fig. 6F illustrates that the mod group had a higher level of collagen *α*1 chain hydroxylation than normal tissue, while the CK treatment effectively inhibited its generation. For instance, the peptide fragment GFPGADGVAGPK showed a specific hydroxylated Pro485 signal in the mod group compared to the con or CK group, indicating an additional *m*/*z* of 16 Da added to Pro485 (Fig. 6G).

Additionally, the immunohistochemical analysis revealed an intensified merged yellow signal in the mod group for VWF (pseudo green) and collagen (pseudo red), and drug intervention group significantly reduced the exposure phenomenon of collagen, as depicted in Fig. 6H (Left panel). The administration of NAC effectively attenuated VWF release by mitigating local bleeding, leading to reduced fluorescence co-localization. Differently, CK impeded the adhesion between VWF and collagen, also resulting in a decreased Pearson coefficient value (Fig. 6H, Left panel). Finally, improvement in blood circulation was assessed by monitoring blood indicators, including RBC aggregation index, hematocrit (HCT), and platelet count (PLT) (Fig. 6I–K). As expected, all of these parameters increased in the mod group, which indicated blood circulation disorders, elevated blood viscosity, and enhanced platelet aggregation. Moreover, abnormalities were found in the mean platelet volume (MPV) (Fig. 6L). However, CK treatment ameliorated these blood indicators by alleviating circulatory issues and inhibiting local thrombosis formation.

### 3.7 CK alleviates DIC in mice by inhibiting platelet adhesion

**Figure 7:**
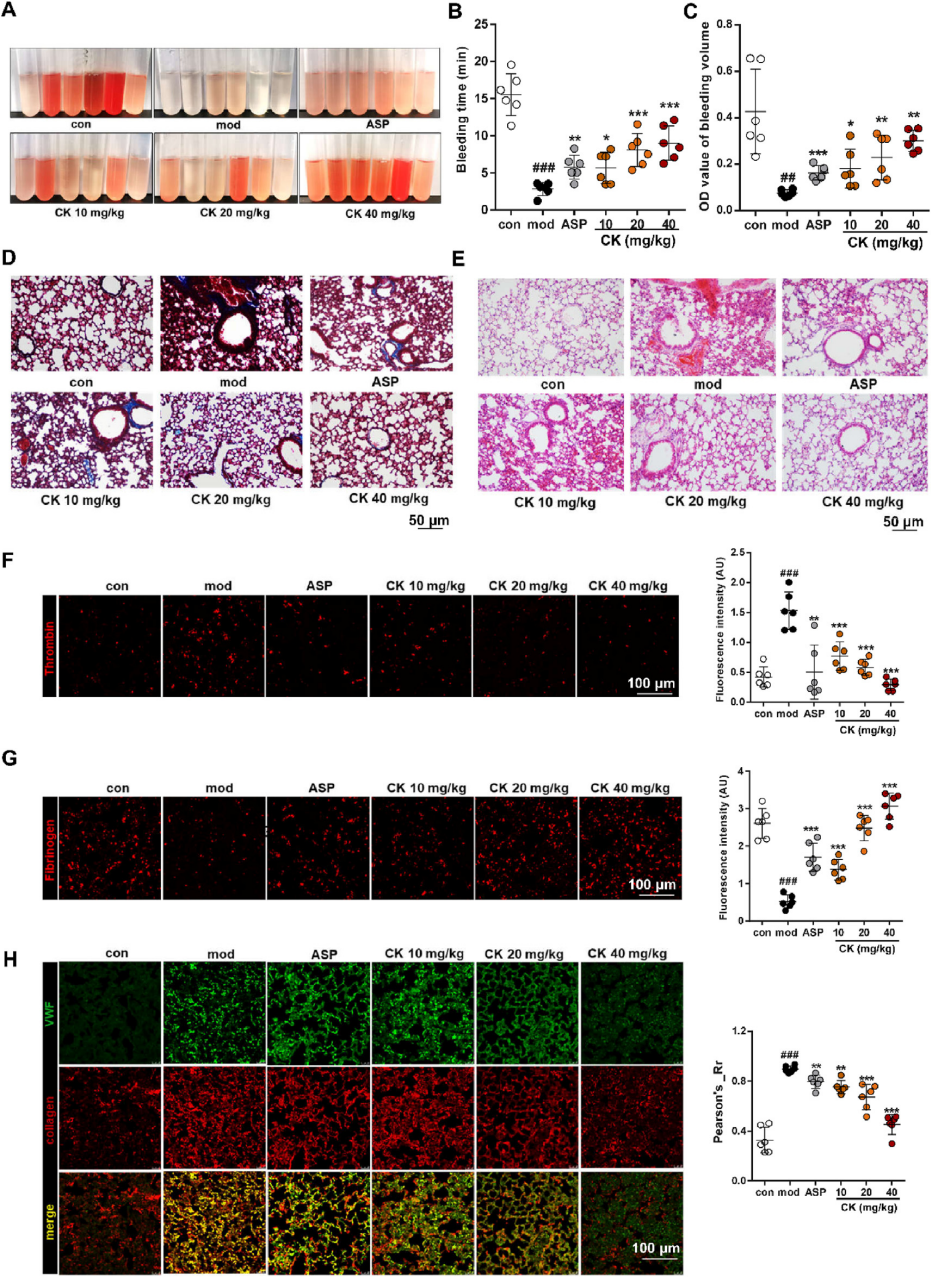
CK inhibits pulmonary thrombus in mouse DIC model. (A) Hemorrhage illustration in the mouse tail in the DIC model. (B, C) Measurement of bleeding time and volume. (D) Masson staining and (E) H&E staining for pathological analysis of lung tissue. (F, G) Immunofluorescence analysis of thrombin and fibrinogen. (H) Fluorescence co-localization analysis of VWF and collagen protein in the lungs. ^##^*P* < 0.01, ^###^*P* < 0.001 *vs*. con group; ∗*P* < 0.05, ∗∗*P* < 0.01, ∗∗∗*P* < 0.001 *vs*. mod group (*n* = 6).

To further validate the pivotal role of targeting PHD2 in platelet thrombosis, the LPS-induced mouse DIC model was utilized to evaluate tail bleeding and thrombus formation in the lung tissue. In Fig. 7A, our investigation of mouse tail bleeding across groups revealed that the mod group had significantly reduced bleeding due to severe DIC. However, treatment with ASP and different doses of CK (10, 20, and 40 mg/kg) improved bleeding. Fig. 7B and C provide additional evidence by the quantifying bleeding time and volume. Histopathological analysis of lung tissue, conducted by Masson (Fig. 7D) and H&E staining (Fig. 7E), also revealed different degrees of thrombosis improvement within each treatment group. The degree of thrombin activation, a key marker for thrombosis, improved dose-dependent with CK intervention (Fig. 7F), as did the conversion of fibrinogen into fibrin (Fig. 7G). Furthermore, VWF-collagen localization assessment revealed significant overlap in thrombus locations within mod lung tissue. However, CK treatment successfully disrupted VWF-collagen localization, reducing thrombosis (Fig. 7H). The results indicate that CK can prevent VWF-collagen interaction, reduce platelet adhesion and hinder the initial stage of platelet thrombosis.

### 3.8 Targeting PHD2 provides a new solution for inhibiting thrombus

**Figure 8:**
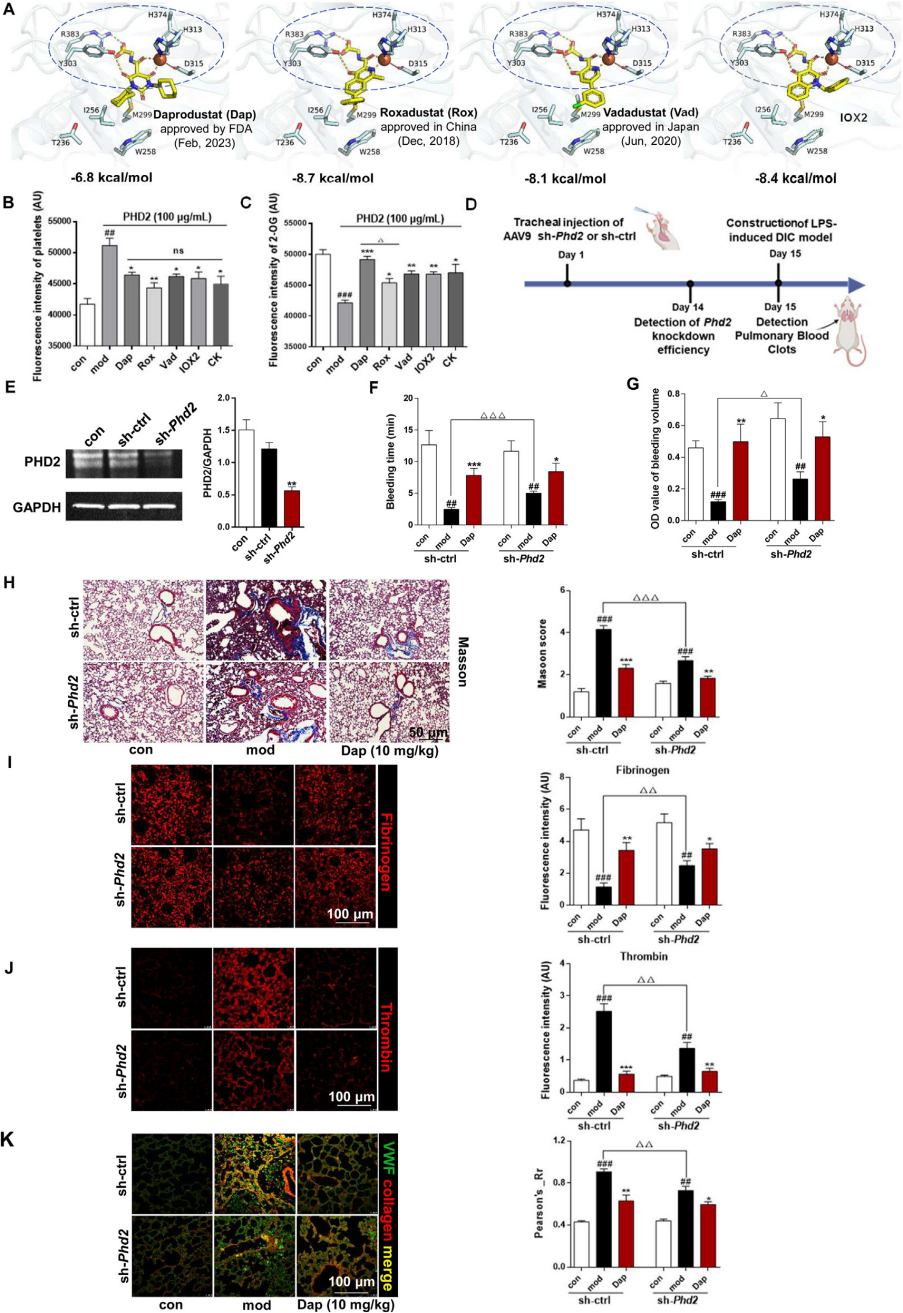
Construction of sh-*Phd2* mice confirms that PHD2 is a potential target for inhibiting platelet adhesion and thrombus. (A) Molecular docking of PHD2 inhibitors Rox, Vad, Dap and IOX2 with PHD2 protein, and dashed lines indicate catalytic pocket. (B) Effect of PHD2 inhibitors on platelet adhesion; ns, multiple comparisons test no significant (*n* = 3). (C) Determination and analysis of the enzymatic activity of PHD2 in the presence of PHD2 inhibitors. ^##^*P* < 0.01, ^###^*P* < 0.001 *vs*. con group; ∗*P* < 0.05, ∗∗*P* < 0.01, ∗∗∗*P* < 0.001, *vs*. mod group; ^Δ^*P* < 0.05, Dap *vs*. Rox group (*n* = 3). (D) Construction scheme of sh-*Phd2* knockdown mice. (E) Western blot analysis for assessing the knockdown efficiency of PHD2 in the sh-*Phd2* group. ∗∗*P* < 0.01, *vs*. sh-con, (*n* = 3). (F, G) Measurement of tail bleeding time and volume of DIC model mice. (H) Masson staining for thrombus analysis of lung tissue. (I, J) Fibrinogen and thrombin immunofluorescence analysis and statistics. (K) Fluorescence co-localization analysis of VWF and collagen in lung tissue. ^##^*P* < 0.01, ^###^*P* < 0.001, *vs*. con group; ∗*P* < 0.05, ∗∗*P* < 0.01, ∗∗∗*P* < 0.001 *vs*. mod group; ^Δ^*P* < 0.05, ^ΔΔ^*P* < 0.01, ^ΔΔΔ^*P* < 0.001, *vs*. mod group in sh-*Phd2* group (*n* = 6).

To study the role of targeting PHD2 in platelet aggregation, we used three PHD2 inhibitors, Dap, Rox, and Vad, which have been clinically approved for treating symptomatic anemia in chronic kidney disease patients^32^, ^33^, ^34^. The structural composition of all these compounds mimics the natural cofactor 2-OG of PHD2's substrate^28^. Molecular docking analysis revealed the interaction between the inhibitors and PHD2's catalytic pocket, where Fe(II) coordinates with the triad of 2-His-1-Asp residues (H374, H313, and D315), exhibiting binding affinities of −6.8, −8.7, and −8.1 kcal/mol respectively (Fig. 8A). The commercially available inhibitor IOX2 also exhibited identical effects (−8.4 kcal/mol). Subsequently, we conducted a study to assess the effect of these inhibitors on platelet adhesion and indirectly measured PHD2 activity by quantifying the remaining 2-OG substrate^35^. The results indicate that all inhibitors, as well as CK (1 μmol/L), effectively reduce platelet adhesion (Fig. 8B) with no significant differences observed. In the assessment of PHD2 activity, CK shows no difference in effect compared to the inhibitors, while Dap exhibits superior inhibitory effect among the inhibitors, thus selected for further investigation (Fig. 8C).

To ascertain the role of PHD2 in platelet thrombus formation, we generated sh-*Phd2* knockdown mice targeting lung tissue (Fig. 8D). After two weeks interference, the sh-*Phd2* group exhibited a significant reduction of approximately 60% in PHD2 expression (Fig. 8E). Fig. 8F and G compare tail bleeding time and bleeding volume between sh-con and sh-*Phd2* groups in the LPS-induced mouse DIC model. The experiments demonstrated a pronounced reduction in tail bleeding upon LPS treatment in the sh-con group. However, the sh-*Phd2* group exhibited a significantly augmented bleeding response, thereby impeding thrombus formation. When PHD2 knockdown mice were treated with Dap (10 mg/kg), the antithrombotic therapeutic effect was also diminished compared with the sh-con group.

It has been observed that both Dap and PHD2 knockdown have resulted in significant improvements in the formation of lung thrombus. It was confirmed through pathological analysis of lung tissues using Masson coupled with H&E staining (Fig. 8H, and Supporting Information Fig. S3). The immunofluorescence tests provided further confirmation of the findings through fibrinogen and coagulation factor thrombin (Fig. 8I and J). Moreover, the evaluation of VWF-collagen localization revealed a substantial overlap in the sh-con mod group. However, both inhibitor and sh-*Phd2* groups disrupted VWF-collagen protein colocalization, thereby reducing lung thrombus formation (Fig. 8K). The above results were also confirmed in the CK-intervention sh-*Phd2* mouse model (Supporting Information Fig. S4). The evidences gathered so far suggests that targeting PHD2 plays a crucial role in platelet thrombus formation.

### 3.9 PHD2 inhibitor combined with ASP enhances antithrombotic effect

**Figure 9:**
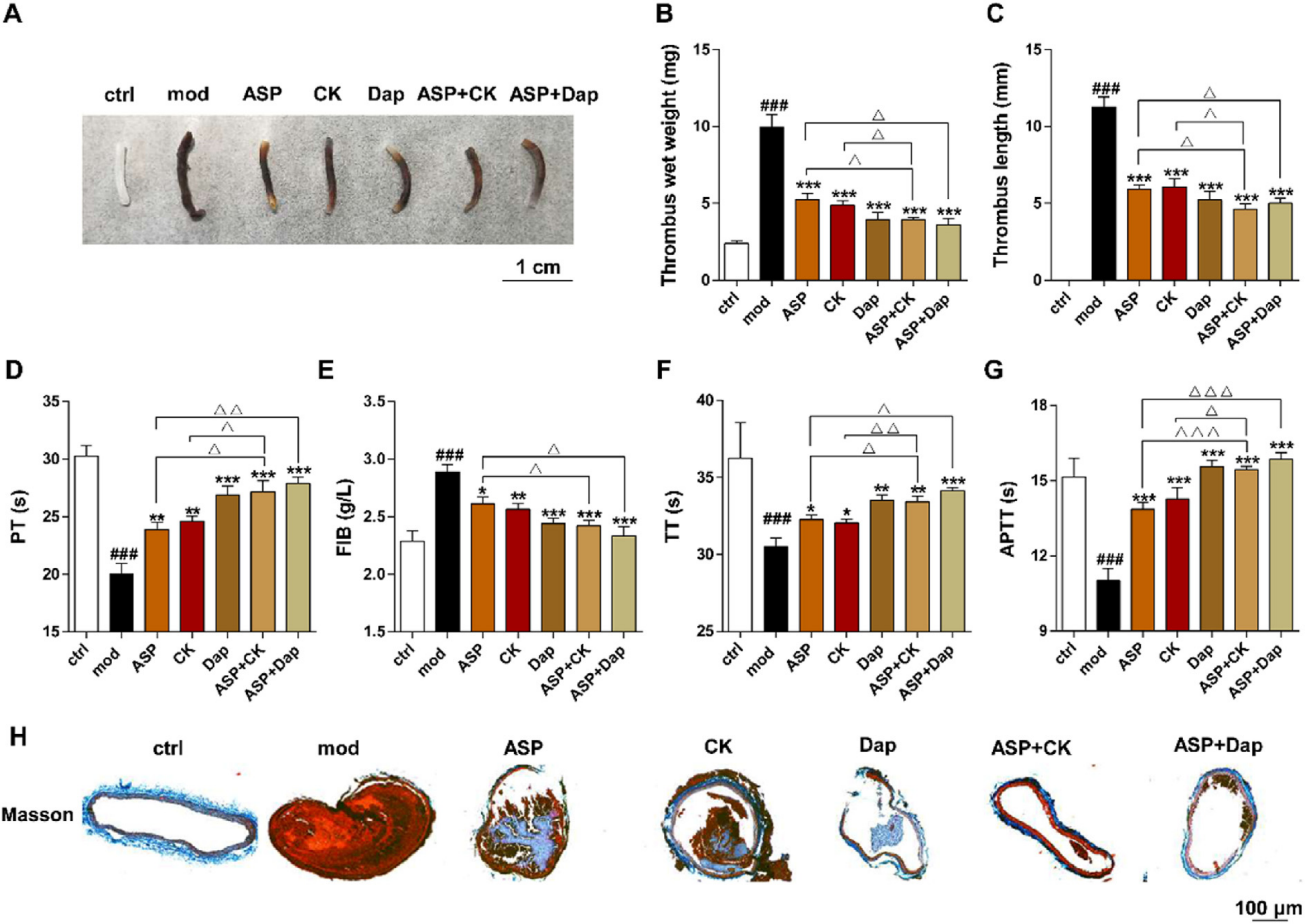
Effect of PHD2 inhibitor combined with ASP on FeCl_3_-induced arterial thrombosis. (A) Occlusive carotid artery thrombus images. (B) Effect of PHD2 inhibitors in combination with ASP on thrombus wet weight and (C) thrombus length. (D–G) Analysis of key coagulation parameters in plasma including PT, APTT, TT, and FIB. (H) Analysis of arterial thrombus histopathology by Masson staining. ^###^*P* < 0.001, *vs*. ctrl group; ∗*P* < 0.05, ∗∗*P* < 0.01, ∗∗∗*P* < 0.001 *vs*. mod group; ^Δ^*P* < 0.05, ^ΔΔ^*P* < 0.01, ^ΔΔΔ^*P* < 0.001, *vs*. respective monotherapy group (*n* = 6).

In addition, we investigated the effects of PHD2 inhibitors combined with antithrombotic drugs on the FeCl_3_-induced carotid artery model. Fig. 9A shows that FeCl_3_ stimulation significantly induced severe occlusive thrombus formation in the carotid artery. The intervention groups showed a noticeable reduction in thrombus formation compared to the model group. The measurements of thrombus wet weight (Fig. 9B) and length (Fig. 9C) indicate that ASP, CK, and Dap, whether administered alone or in combination, exhibited sound antithrombotic effects. Furthermore, the combined use of ASP and CK displayed superior efficacy compared to their individual use, and similar effects were observed with the combination of Dap and ASP.

Results for representative coagulation indicators, including Prothrombin Time (PT), Activated Partial Thromboplastin Time (APTT), Thrombin Time (TT), and FIB (Fibrinogen), in Fig. 9D–G, demonstrate that the presence of thrombus significantly decreased PT, APTT, and TT, while increasing FIB content. All treatment groups showed improvement when compared to the mod group. Masson staining of thrombi in Fig. 9H revealed significant arterial intimal injury and dense structure in the mod group, leading to severe occlusive thrombi. However, the intervention groups exhibited less intimal damage, looser thrombus structure, and a significant reduction in thrombus surface area. In conclusion, combining CK or PHD2 inhibitors with aspirin shows more pronounced therapeutic effects on thrombus formation than individually, providing insights for treating thrombosis.

## 4 Discussions

**Figure 10:**
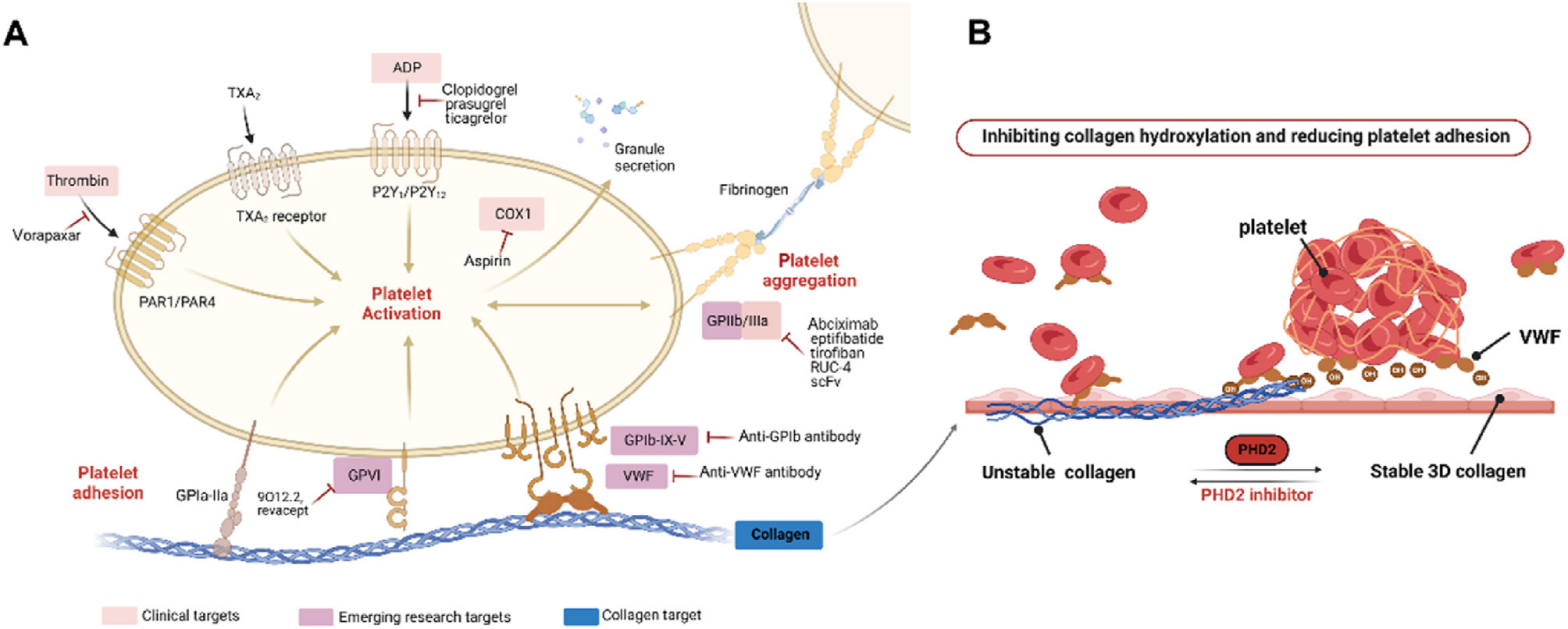
The schematic diagram for antiplatelet adhesion by regulating the three-dimensional structure of collagen. (A) Targets for antiplatelet thrombosis with current research. Light red shading indicates clinically approval drug targets, dark red highlighting denotes newly emerging tactics, and blue representing the collagen target illuminated in this paper. (B) The mechanism diagram targets the PHD2 protein to modulate the three-dimensional structure of collagen for inhibiting platelet adhesion.

Platelets play a crucial role in the process of hemostasis and thrombus formation. Controlled activation and aggregation of platelets are essential to achieve hemostasis at sites of vascular injury. However, uncontrolled platelet activation can lead to the formation of pathological thrombi, which can cause circulatory disorders like DIC, myocardial infarction, and stroke^36^. The current targets of antiplatelet therapies are illustrated in Fig. 10A. There are four primary classes of antiplatelet drugs utilized in clinical practice, individually or in combination, which target different pathways involved in platelet activation^3^. These include cyclooxygenase 1 (COX1; also known as PTGS1) inhibitors like aspirin^37^, ADP P2Y12 receptor inhibitors such as clopidogrel, prasugrel, and ticagrelor^38^, PAR1 antagonists like vorapaxar^39^, and GPIIb/IIIa inhibitors like abciximab, eptifibatide, and tirofiban^40^. Cilostazol is another FDA-approved antiplatelet drug that improves peripheral vascular diseases and reduces morbidity and mortality associated with arterial thrombosis^41^. Although these drugs are effective, they can cause bleeding risks that may lead to gastric ulcers, prolonged bleeding, aplastic anemia, and thrombocytopenic purpura. Moreover, a significant proportion of the population develops resistance to these drugs, necessitating the development of safer and more effective therapeutic approaches with minimal side effects^42^. In recent years, several innovative antiplatelet therapies have emerged in preclinical and early clinical trials, demonstrating the potential to inhibit thrombus formation while maintaining hemostasis. These therapeutic target phosphoinositide 3-kinase *β* (AZD6482)^43^, protein disulfide isomerase (ML359)^44^, activated GPIIb/IIIa with inward signaling modulation (RUC-4, scFv)^45^^,^^46^. And inhibitors targeting the platelet GPVI-mediated adhesion pathway (9O12.2, revacept), indicating that inhibition of platelet adhesion to collagen is closely related to subsequent platelet activation^47^^,^^48^.

In the process of platelet thrombosis, the interaction between platelet collagen receptors such as GpVI, *α*2*β*1, or the GpIb/VWF complex and subendothelial collagen structures is critical in forming platelet-rich thrombi that obstruct the vascular system. Recently, there has been a shift towards innovative therapeutic targets, with a specific focus on inhibitors that target the GPIb–VWF axis. The GPIb–IX–V complex binds to VWF at sites of vascular injury through the GPIb subunit, which binds to collagen, thereby initiating platelet adhesion and thrombus formation. Various inhibitors targeting the GPIb–VWF axis have been developed, including anti-GPIb or anti-VWF antibodies^49^^,^^50^, GPIb antagonists derived from snake venom^51^, and recombinant fragments of GPIb or VWF^52^^,^^53^. However, some development efforts have been halted due to an increased risk of hemorrhaging associated with their usage. Furthermore, the three-dimensional architecture of collagen also exerts a significant influence on its interaction with VWF, unfortunately, this crucial aspect has been disregarded for a substantial duration.

In recent years, there has been an increasing trend in developing innovative drugs by elucidating the mechanisms behind the active constituents of TCM using traditional wisdom. In this study, we found that CK within *P. notoginseng* saponins plays a pivotal role in improving platelet adhesion dysfunction (Supporting Information Fig. S5), while predicting PHD2 as a key therapeutic target. The insights in this paper highlight that targeting the structural framework of collagen by suppressing proline hydroxylation to inhibit the VWF-collagen interaction could potentially provide an alternative solution for preventing platelet thrombus formation (Fig. 10B).

Previous PHD2 inhibitor drugs target the catalytic pocket^28^, Rox (IC_50_ = 117.2 nmol/L), Vad (IC_50_ = 256.9 nmol/L) and Dap (IC_50_ = 140.8 nmol/L), primarily targeted the HIF-1*α* signaling pathway to stabilize HIF-*α* and enhance EPO expression, mainly for conditions such as anemia. Their investigations into the involvement of PHD2 in collagen hydroxylation are limited. A few antiplatelet drug only focused on evaluating collagen hydrogel applications for wound dressings^54^. Hence, our study broadens the potential therapeutic applications of clinically approved PHD2 inhibitors, potentially serving as promising drug candidates for addressing platelet agglutination and maintaining circulatory homeostasis, and PHD2 inhibitors have shown promising effects in the treatment of atherosclerosis, as detailed in the supporting information (Supporting Information Figs. S6 and S7). Furthermore, molecular docking revealed that CK not only competitively targets the catalytic pocket of PHD2 but also occupies the substrate binding pocket, involving specific amino acid residues (T236, I256, W258, and M299). Notably, CK exhibits a comparable IC_50_ value of approximately 209.8 nmol/L (Supporting Information Fig. S8). It may serve as a valuable reference for the drug design targeting the PHD2 structure. Although the binding of VWF to subendothelial collagen has been elucidated, a comprehensive understanding of the precise mechanisms governing platelet adhesion and activation for aggregate formation through collagen prolyl hydroxylation remains elusive, necessitating further investigation.

## 5 Conclusions

The targeted inhibition of PHD2 to attenuate collagen hydroxylation and subsequently impede VWF binding to collagen represents a promising innovative antiplatelet strategy for effectively preventing pathological thrombus formation while ameliorating blood circulatory disorders. However, the novel antiplatelet treatment paradigms proposed in this paper require further evaluation in clinical practice. As the comprehension of thrombosis and anticoagulation mechanism continues to progress, safe and efficacious drugs will be provided for expanding patient population.

## Author contributions

Chuanjing Cheng: Writing – original draft, Validation, Methodology, Formal analysis, Data curation. Kaixin Liu: Methodology. Jinling Zhang: Methodology. Yanqi Han: Methodology. Tiejun Zhang: Methodology. Yuanyuan Hou: Writing – review & editing, Conceptualization. Gang Bai: Writing – review & editing, Funding acquisition, Conceptualization.

## Conflicts of interest

The authors declare no conflicts of interest.

## Appendix A Supplementary data

The following is the Supporting Information to this article:
