# Supplementary material for: Ginsenoside CK targets PHD2 to prevent platelet adhesion and enhance blood circulation by modifying the three-dimensional arrangement of collagen
Source: Acta Pharm Sin B. 2024 Dec 31;15(3):1497–513. doi: 10.1016/j.apsb.2024.12.038 (PMC12069249; doi:10.1016/j.apsb.2024.12.038)
Supplement: Multimedia component 1 [file mmc1.pdf]

Supporting Information for

Original article

Ginsenoside CK targets PHD2 to prevent platelet adhesion and enhance blood circulation by modifying the three-dimensional arrangement of collagen

Chuanjing Cheng[a], Kaixin Liu[a], Jinling Zhang[a], Yanqi Han[b], Tiejun Zhang[b], Yuanyuan Hou[a,*], Gang Bai[a,*]

[a]*State Key Laboratory of Medicinal Chemical Biology, College of Pharmacy and Tianjin Key Laboratory of Molecular Drug Research, Nankai University, Tianjin 300353, China*

[b]*State Key Laboratory of Drug Delivery Technology and Pharmacokinetics, Tianjin Key Laboratory of Quality markers of Traditional Chinese medicine, Tianjin Institute of Pharmaceutical Research, Tianjin 300462, China*

Received 10 July 2024; received in revised form 28 October 2024; accepted 26 November 2024

*Corresponding authors.

E-mail addresses: houyy@nankai.edu.cn (Yuanyuan Hou), and gangbai@nankai.edu.cn (Gang Bai).

1. LC–MS analysis method of metabolomics

LC–MS analysis method and metabolite identification process refer to previous report[1]. In the nontargeted metabolomics analysis, the samples were analyzed by AB Triple TOF 6600 mass spectrometers (AB SCIEX, USA) coupled to an Agilent 1290 Infinity LC liquid chromatography system (Agilent, USA). For LC separation, ACQUITY UPLC BEH Amide column (2.1 mm × 100 mm i.d., 1.7 µm) was use. 5 µL sample was injected and separated with a 12 min gradient starting at 5% buffer A (25 mmol/L CH_3COONH_4 and 25 mmol/L ammonium hydroxide in water) and 95% buffer B (100% MeCN) stayed for 0.5 min. The buffer A followed by a stepwise increase to 35% in 7 min. Then, the buffer A linear changed to 60% in 8 min and stayed for 1 min. The buffer A followed by a decrease to 5% in 9.1 min and stayed for 3.9 min. The electrospray ionization mass spectra were acquired in positive and negative ion mode, respectively.

Information dependent acquisition (IDA) was used to collect full scan MS and MS/MS information simultaneously. The ion spray voltage was set to ±5500 V for positive and negative mode, declustering potential (DP) was set to ±60 V for positive and negative mode, and the heated-capillary temperature was maintained at 600 °C. The curtain gas flow, nebulizer and heater gas were set to 30, 60 and 60 arbitrary units, respectively. The LC–MS chromatograms were shown in Fig. S1.

A

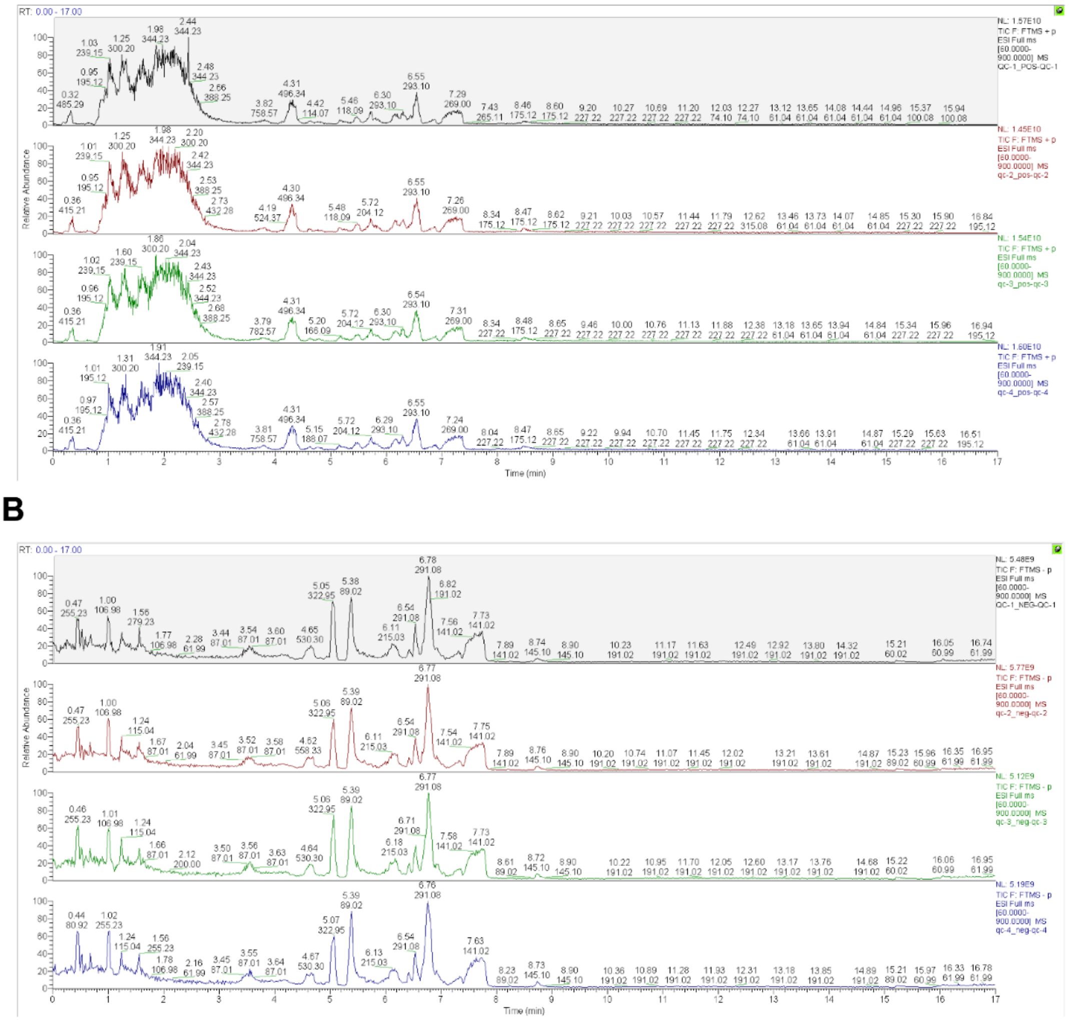

Figure S1 The LC–MS chromatograms of nontargeted metabolomics analysis. The total ion chromatogram (TIC) of positive mode (A) and in negative mode (B).

2. Drug affinity responsive target stability (DARTS)

To ascertain the target of ginsenoside CK, we employed DARTS technique following

the established protocol[2]. Briefly, damaged soft tissues were lysed and subjected to reaction using TNC buffer (50 mmol/L Tris-HCl, 50 mmol/L NaCl, 10 mmol/L $CaCl_2$). Subsequently, lysate was digested at a 1:3000 ratio with pronase (MedChemExpress, HY-114158A) at 25 °C for 3 min, followed by SDS-PAGE analysis. Finally, differential proteins were identified through Western blot analysis or HPLC–MS/MS analysis (outsourced to Beijing Protein Innovation) for target determination.

3. Expression and purification of recombinant PHD2 protein

The DNA sequence encoding PHD2 protein (UniProtKB-Q9GZT9, 181-426aa) was cloned into the pET28a(+) vector. Recombinant plasmids were introduced into *Escherichia coli* BL21 strain and cultured at 37 °C in LB medium supplemented with ampicillin (100 μg/mL). Upon reaching an optical density of 0.6, protein expression was induced using 1 mmol/L isopropyl *β*-D-1-thiogalactopyranoside at 18 °C for 18 h. Subsequently, cells were harvested and subjected to 30 min ultrasound treatment at 300 W in an ice bath, and the resulting supernatant was loaded onto a nickel column (GE Healthcare). 40 mmol/L imidazole was used for impurity removal, and 400 mmol/L imidazole was applied for eluting PHD2 protein. Protein concentration was achieved through centrifugal filtration with a molecular weight cutoff of 3000 Da. Finally, SDS-PAGE analysis confirmed protein purity, and all proteins were concentrated and stored in PBS at –80 °C.

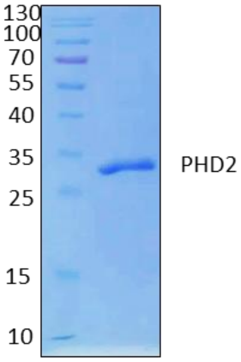

Figure S2 SDS-PAGE assay of expressed WT PHD2 protein.

4. Surface plasmon resonance (SPR) analysis

The interaction between ginsenoside CK and PHD2 was investigated through SPR experiments conducted using a Biacore T200 optical biosensor (GE Healthcare, Pittsburgh, PA, USA). The recombinant PHD2 protein (50 μg/mL) was immobilized on a sodium acetate buffer (pH 4.5) surface. In each binding cycle, ginsenoside CK solution (31.3–50 μmol/L) was injected at a flow rate of 30 μL/min for 1 min, followed by a 150-s dissociation monitoring phase. Data collection and organization were carried out in accordance with standardized procedures.

5. Microscale Thermophoresis (MST)

A Monolith NT.115 machine from Nano Temper (Munich, Germany), was used to do MST measurements. For labeling, we used the L001 Monolith NT.115 protein labeling kit with the RED-NHS dye on the purified PHD2 or collagen protein, making it a concentration of 200 nmol/L. The mixture was put into a water-loving silicon tube (K004 Monolith NT.115). We analyzed the data using the Monolith software.

6. Fluorescence Thermal Shift Assay (FTSA)

Initially, we combined 10 μmol/L PHD2 (with or without 10 μmol/L ginsenoside CK) and assay buffer [1× PBS (pH 7.4)]. Then, we added Thermal Shift Dye Kit™ to reach a 20 μL final volume, loading it into a 96-well PCR plate. Subsequently, the samples were heated in a PCR system (StepOnePlus, Applied Biosystems) at a rate of 1 °C per minute, maintaining 37 °C for 74 cycles. Fluorescence was monitored at 533 nm excitation and 572 nm emission wavelengths. Reference wells were utilized for melting temperature (T_m) comparisons. T_m values were analyzed using the Protein Thermal Shift software, LightCycler® 96.

7. H&E analysis of sh-PHD2 mouse DIC model by daprodustat (Dap)

It has been observed that both Dap and PHD2 knockdown have resulted in significant improvements in the formation of lung thrombus. To determine its histopathological improvement, H&E staining was performed on paraffin sections of lung tissue with

reference to conventional staining methods (Fig. S3). It was consistent with the result of Masson coupled with H&E staining on lung tissues (Fig. 8H).

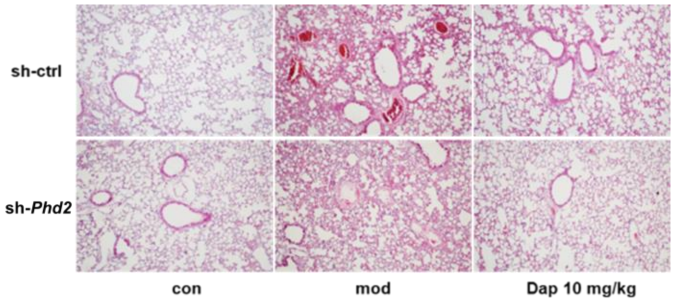

Figure S3 H&E staining analysis of the effect of thrombus in lung tissue of sh-*Phd2* mice. Figure S3 uses the same con and mod groups for data analysis as Figure S4D.

8. Effect of CK on DIC model in sh-*Phd2* mice

The comparative analysis of CK administration for tail bleeding time and bleeding volume under the LPS-induced mouse DIC model (Fig. S4A–S4C). The experiments were employed both in normal control mice (sh-ctrl) and PHD2 knockout mice (sh-*Phd2*). The results reveal that when the sh-con group was treated with LPS, there was a significant reduction in tail bleeding. However, the sh-*Phd2* group displayed a much larger increase in bleeding and significantly inhibited thrombus formation. When PHD2 knockout mice were treated with CK (20 mg/kg), the antithrombotic therapeutic effect was also affected compared with the sh-crtl group. The result has been observed that both CK and PHD2 knockdown have resulted in significant improvements in the formation of lung thrombus.

Moreover, the similar result was confirmed through pathological analysis of lung tissues using H&E and Masson staining (Fig. S4D and S4E). The immunofluorescence tests further validated the findings for coagulation factor fibrinogen and thrombin (Fig. S4F and S4G). Evaluation of VWF-collagen localization revealed a substantial overlap of thrombi in the sh-ctrl group induced by LPS. However, both CK administration and sh-*Phd2* knockout disrupted VWF-collagen protein localization, thereby reducing lung thrombus formation (Fig. S4H).

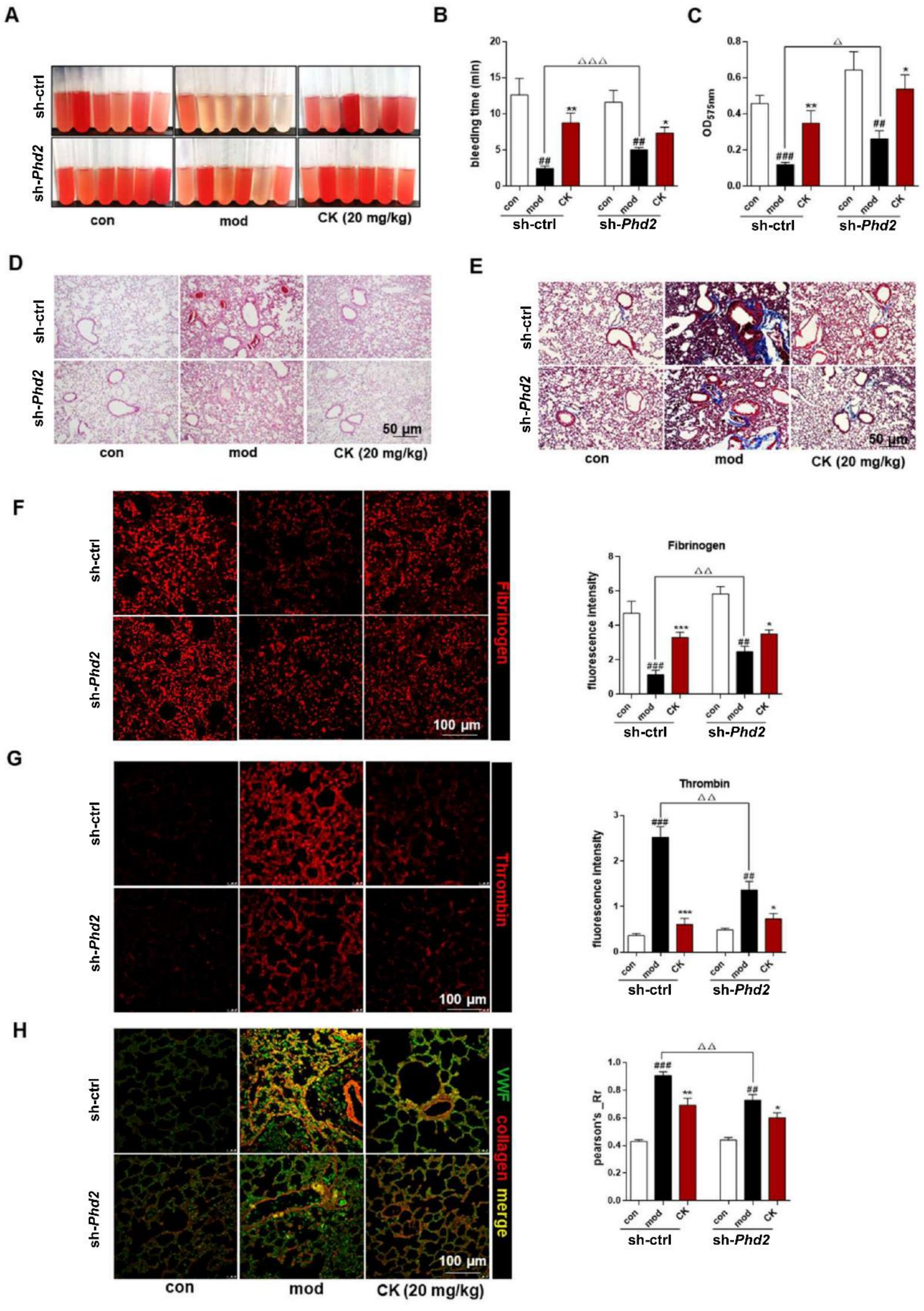

Figure S4 Effect of CK on DIC-induced platelet adhesion and thrombosis in sh-PHD2 mice. (A) Hemorrhage illustration in the mouse tail in sh-*Phd2* mice. (B, C) Measurement of bleeding time and volume. (D, E) H&E and Masson staining for

thrombus analysis of lung tissue. (F, G) Fibrinogen and thrombin immunofluorescence analysis and statistics. (H) Fluorescence co-localization analysis of VWF and collagen in lung tissue. $^{##}P < 0.01$, $^{###}P < 0.001$, vs. con group; $^{*}P < 0.05$, $^{**}P < 0.01$, $^{***}P <$ 0.001 vs. mod group; $^{\triangle}P < 0.05$, $^{\triangle\triangle}P < 0.01$, $^{\triangle\triangle\triangle}P < 0.001$, vs. mod group in sh-*Phd2* group ($n = 6$). Figure S4D uses the same con and mod groups for data analysis as Figure S3, and S4E–S4H uses the same con and mod groups for data analysis as Figures 8H-8K, respectively.

9. Evaluation of the activity of representative saponins in *Panax notoginseng*

The figure below illustrates the inhibitory activity of representative 16 kind saponins derived from *Panax notoginseng* on platelet adhesion, as well as their inhibitory efficiency on the PHD2 enzyme (Fig. S5). These prototype saponins and its metabolites were procured from Shanghai Yuanye Biotechnology Co., Ltd. (Shanghai, China), which named ginsenoside Rg1 (B21057), Rg2 (B21058), Rg3 (B21059), Re (B21055), Ro (B21068), Rd (B21054), Rb1 (B21050), Rh1 (B21061), Rh2 (B21062), F1 (B21046), F2 (B21047), CK (B21045), 20(*S*)-protopanaxatriol (PPT, B21620), 20(*S*)-protopanaxadiol (PPD, B21619), Notoginsenoside R1 (NR1, B21099), respectively. Detection of platelet adhesion was described in the Materials and Methods section 2.8. The inhibitory activity on PHD2 enzyme activity detection method was determined by the following supporting information 10. It is evident that there are variations in the activity levels among different saponins, with CK demonstrating prominent excellence in both inhibition rate testing (>0.2). Therefore, Ginsenoside CK has been selected as the representative active compound to elucidate its mechanisms underlying platelet adhesion inhibition and improvement of blood circulation.

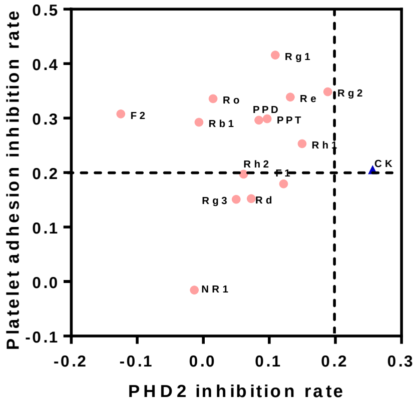

Figure S5 Evaluation *Panax notoginseng* saponins anti-platelet adhesion and PHD2 inhibition activity.

10. The effect of CK on atherosclerosis induced by combined treatment with AAV-PCSK9 and high-fat diet, along with partial carotid artery ligation

As reported in the literature (PMID: 28504688), we conducted carotid artery ligation combined with AAV-PCSK9 induction to establish an atherosclerosis model. Specifically, C57 mice were injected via tail vein with AAV-PCSK9 (5×10^{11} VG) and fed a high-fat diet (Cat # D12108C, New Brunswick, NJ, USA). One week after AAV-PCSK9 injection, mice underwent partial carotid artery ligation surgery and continued on the high-fat diet for 4 weeks. Concurrently, Ginsenoside CK and Daprodustat were injected intraperitoneally, with a daily dose of 5 mg/kg. Finally, plasma was collected for lipid profile assessment, the aortic arch and carotid artery were used oil red staining to evaluate the level of atherosclerosis. The experimental process is shown in Fig. S6.

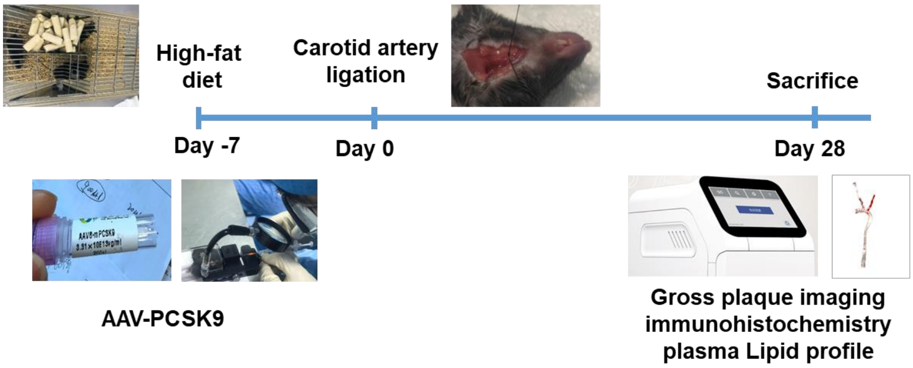

Figure S6 Overall study design and work flow of animal model of atherosclerosis.

The experimental results indicate that partial ligation of the carotid artery accelerated the progression of atherosclerosis in C57 mice treated with AAV9-PCSK9. Hemodynamic disturbances in the left carotid artery (LCA) following ligation precipitated rapid onset of atherosclerosis at that site. As depicted in Fig. S7A, the overall arterial images showed minimal plaque formation in the non-ligated right carotid artery (RCA) and aortic segments, while intervention with CK and Dap drugs significantly reduced plaque formation, notably improving atherosclerosis. Further Oil Red O staining and quantitative analysis of plaque formation at the carotid site (Fig. S7B) demonstrated a significant increase in lesion area and intimal thickening in the model group compared to the control group, effects markedly attenuated by CK and Dap interventions. Additionally, CK treatment contributed to favorable changes in lipid profiles associated with atherosclerosis, including LDL-C, CHOL, TG, HDL-C, HCY, and GLU levels, thereby mitigating the conditions conducive to plaque formation and subsequent thrombosis (Fig. S7C–S7H).

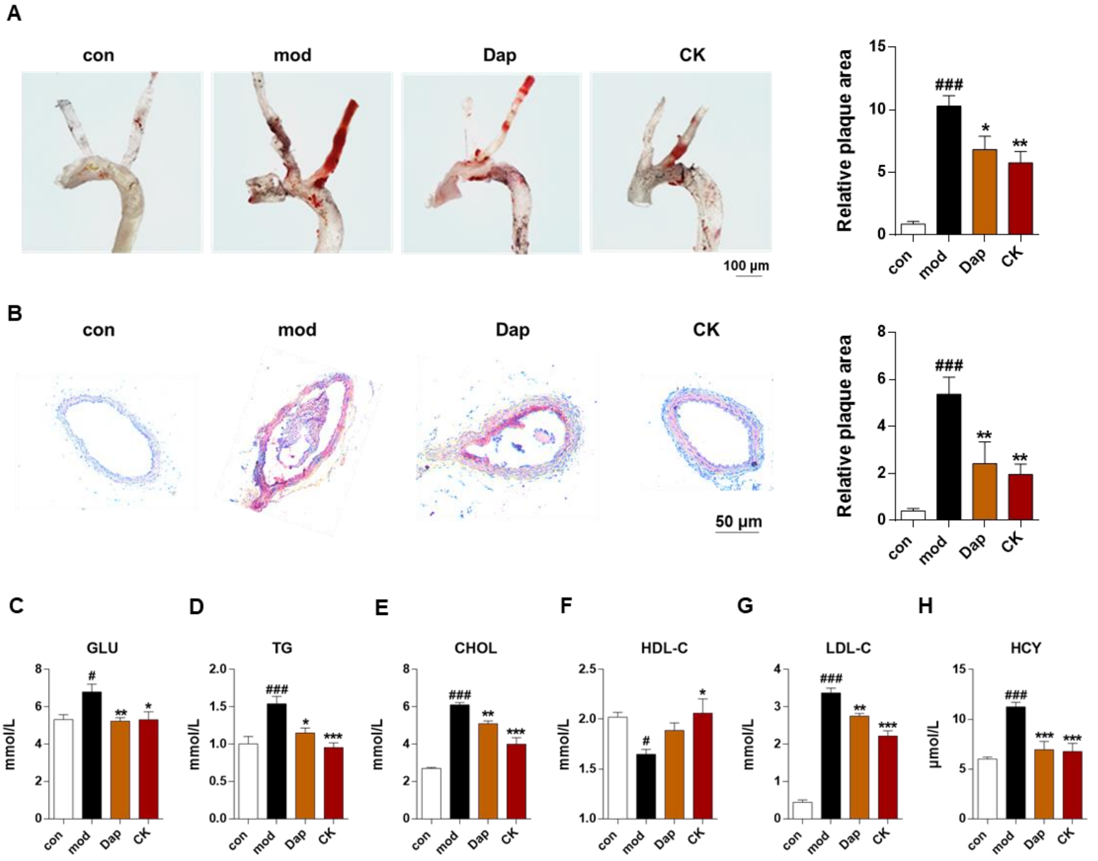

Figure S7 The effect of CK on atherosclerosis induced by combined treatment with AAV-PCSK9 and high-fat diet, along with partial carotid artery ligation (A) Imaging of atherosclerotic plaques. (B) Oil-red-O staining of cross-section of carotid artery on ligated side. (C–H) Plasma lipid profile in C57 animals after AAV-PCSK9 injection and high-fat diet feeding. $^{\#}P < 0.05$, $^{\#\#\#}P < 0.001$, *vs*. con group; $*P < 0.05$, $**P < 0.01$, $***P < 0.001$ *vs*. mod group ($n = 6$).

11. Detection on the inhibitory activity on PHD2 enzyme activity

The inhibition of PHD2 activity was assessed by indirectly measuring the remaining 2-OG substrate, employing established methodologies by PHD2 catalytic 2-OG for quantification as described in published literature with appropriate modification[3]. The effectiveness of PHD2 inhibitors was assessed by indirect quantification of residual 2-OG. After preincubating the PHD2 enzyme with different concentrations of CK (ranging from 0.01 to 10 μmol/L) for 12 h, the reaction was started by adding *o*-phenylenediamine under 0.5 mol/L hydrochloric acid. The fluorescence intensity generated by the interaction between the remaining 2-OG and *o*-phenylenediamine in the reaction system was measured to determine the inhibitory effect of CK on PHD2.

The results demonstrated that the series of diluted CK (10, 1, 0.1, 0.01 μmol/L) effectively inhibited the substrate 2-OG utilization efficiency of PHD2. The IC_{50} value was about 209.8 nmol/L ($n = 3$).

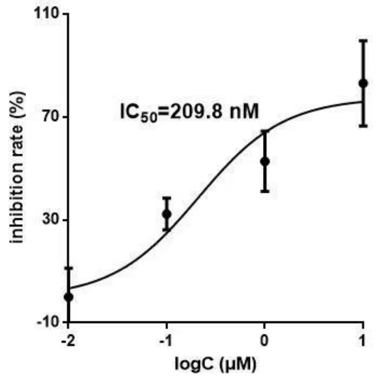

Figure S8 Effect of CK on inhibition efficiency of PHD2 enzyme activity.

References

[1] Blaženović I, Kind T, Ji J, Fiehn O. Software tools and approaches for compound identification of LC–MS/MS data in metabolomics. *Metabolites* 2018;**8**:31.

[2] Lomenick B, Hao R, Jonai N, Chin RM, Aghajan M, Warburton S, et al. Target identification using drug affinity responsive target stability (DARTS). *Proc Natl Acad Sci USA* 2009;**106**:21984-9.

[3] McNeill LA, Bethge L, Hewitson KS, Schofield CJ. A fluorescence-based assay for 2-oxoglutarate-dependent oxygenases. *Anal Biochem* 2005;**336**:125–31.
